# Changes in Microbial Community Assemblages Due To Urban Pollution, Detected via rRNA Gene Amplicon Sequencing in the Magdalena River, Mexico City

**DOI:** 10.1007/s00248-025-02580-7

**Published:** 2025-08-02

**Authors:** R. Cruz-Cano, L. Bretón-Deval, M. Martínez-García, P. Díaz-Jaimes, M. Kolb

**Affiliations:** 1 Instituto de Geografía, Universidad Nacional Autónoma de México, Ciudad Universitaria, Circuito de la Investigación Científica, C.P. 04510 Coyoacán, Mexico City, Mexico; 2 Instituto de Biotecnología, Universidad Nacional Autónoma de México, Cuernavaca, 62210 México; 3 Facultad de Estudios Superiores Iztacala, Universidad Nacional Autónoma de México, Tlalnepantla Estado de México C.P. 54110, Avenida de los Barrios #1, Los Reyes Iztacala, México; 4 Unidad de Ecología y Biodiversidad Acuática, Instituto de Ciencias del Mar y Limnología, Universidad Nacional Autónoma de México, Ciudad Universitaria, Circuito Exterior S/N, C.P. 04510 Coyoacán, Mexico City, Mexico; 5 Posgrado en Ciencias Biológicas, Universidad Nacional Autónoma de México, Ciudad Universitaria, Circuito Exterior S/N, C.P. 04510 Coyoacán, Mexico City, Mexico; 6 Secretaria de Ciencia, Tecnología E Innovación, Crédito Constructor, Benito Juárez, Avenida de los Insurgentes Sur 1582, 03940 Humanidades Ciudad de Mexico, Mexico

**Keywords:** Microbial, Diversity, Pollution, Seasonality, Mexico

## Abstract

As freshwater sources become increasingly polluted and depleted, the delicate balance of aquatic environments is disrupted, leading to cascading effects throughout entire ecosystems. This disruption manifests in various ways, including changes in water chemistry, temperature fluctuations, and the introduction of contaminants, all of which contribute to alterations in microbial communities. We applied eDNA metabarcoding to characterize microbial communities along an anthropogenic pollution gradient in the Magdalena River, a tropical river in Mexico City. Sampling was conducted at four sites representing different levels of human influence. Results revealed differences in both bacterial and microeukaryotic community compositions between sites. In areas with low to moderate disturbance, bacterial genera associated with nitrogen cycling and plant–microbe interactions (e.g., *Rhizobacter*, *Rhodoferax*, and *Flavobacterium*) were predominant, whereas in more heavily impacted sites, genera linked to enteric, nosocomial, or fecal sources (e.g., *Arcobacter*, *Acinetobacter*, and *Aeromonas*) dominated. Peri-urban sites exhibited higher alpha diversity at the phylum level for bacteria, and microeukaryotic communities; two phyla account for over 75% of the relative abundance throughout the year (Ciliophora & Chlorophyta). Statistical analysis showed that water quality influences microbial composition in the sites. These findings demonstrate that urban influence alter microbial community composition, showing similar patterns to other studies. Our study, however, also discovered certain taxa that had not been previously recorded in tropical urban rivers, thereby broadening the existing knowledge, which has primarily been based on temperate systems. This research offers one of the initial thorough evaluations of microbial communities in urban rivers in Mexico and highlights the potential of eDNA metabarcoding as a valuable tool for environmental monitoring.

**Graphical Abstract:**

Changing microbial communities (eukaryotic and prokaryotic) and physicochemical variables along a human influence gradient. Left circles in any of the sites show the characteristic and most abundant prokaryotic groups; right circles show the characteristic and most abundant microeukaryotic groups. The figure also shows the most common groups of bacteria and eukarya over all sites (lower left corner)

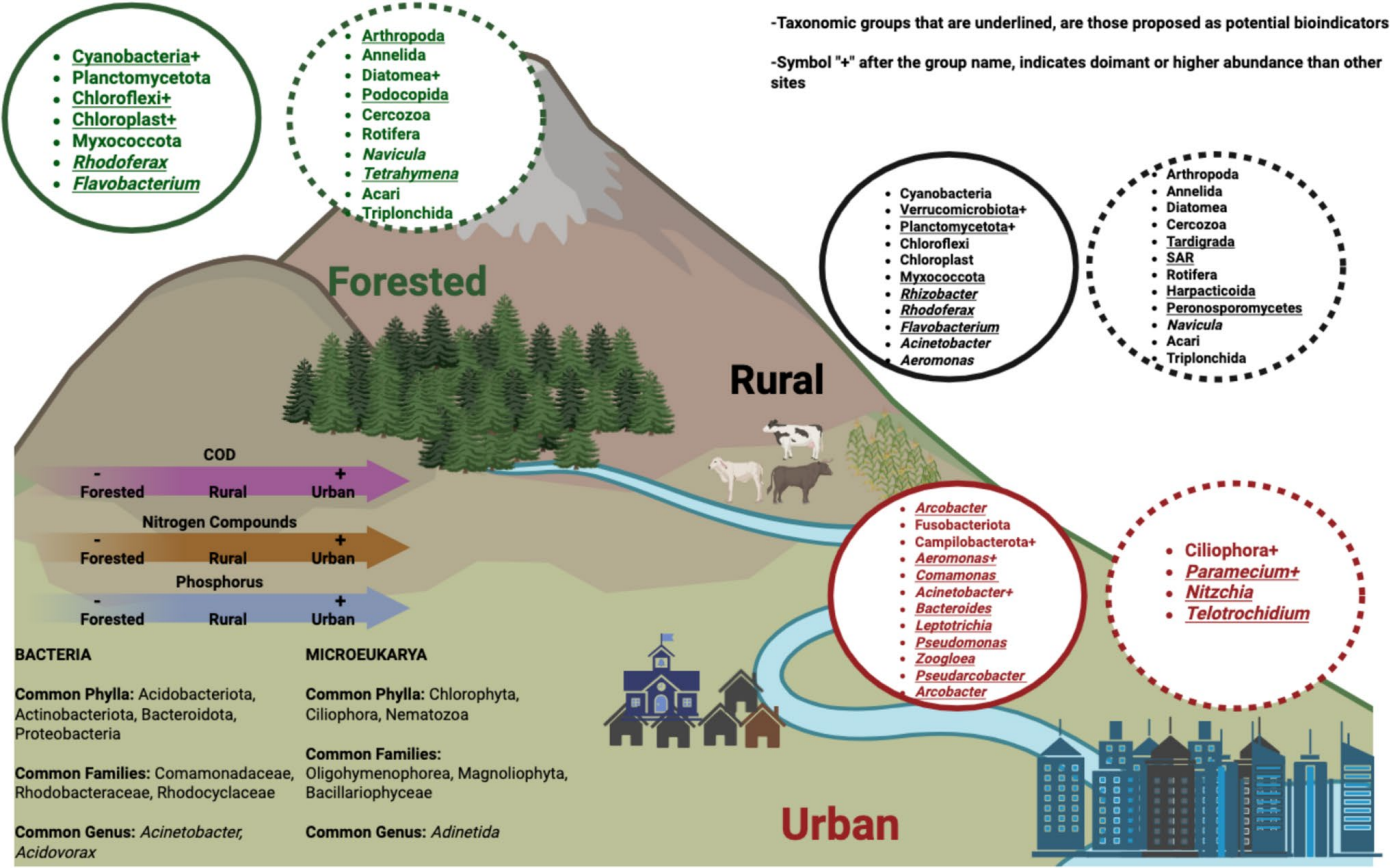

**Supplementary Information:**

The online version contains supplementary material available at 10.1007/s00248-025-02580-7.

## Introduction

Rivers play a crucial role in aquatic ecosystems by supporting biodiversity and providing vital ecosystem services; however, they are increasingly impacted by human activities such as urbanization, industrialization, habitat degradation, agricultural intensification, and climate change [1–3]. Rivers transport water and nutrients to almost every part of the planet and drain approximately 75% of the earth’s surface water, acting as dynamic interfaces between terrestrial and aquatic environments [1, 3]. They act as pathways for the movement of water, energy, and nutrients across ecosystems, landscapes, and regions. While rivers are widely acknowledged for their role in supporting vertebrate biodiversity, it is the microbial communities (comprising archaea, bacteria, fungi, and microeukaryotes) that represent a highly diverse group responsible for a wide range of essential ecosystem functions [4, 5]. These microorganisms drive nutrient cycling through biogeochemical processes and contribute actively in the decomposition of organic matter, and pollutant remediation, making them key drivers of ecosystem functions that influence water quality, food webs, and overall ecosystem stability [1, 4–7].

Although rivers provide numerous benefits for human activities, many tropical urban rivers worldwide are often used as part of sewage infrastructure for industrial effluents and domestic wastewater, which severely compromises water quality [8]. This degradation is frequently accompanied by shifts in the composition, structure, and diversity of microbial communities [9–11]. Notably, changes in microbial community composition, structure, and diversity can serve as early warning indicators of environmental alterations often manifesting before significant effects become observable in larger organisms or broader ecosystem processes [12–15].

In recent years, there has been growing interest in understanding the roles microorganisms play in maintaining ecosystem functions, highlighting their potential as bioindicators [16–22]. One of the main advantages of using microorganisms as sentinels of environmental change lies in their ubiquity, adaptability, and rapid responsiveness to environmental conditions. Traditional methods for studying microbial communities, such as culture-based techniques and direct microscopic observations, are limited in their ability to provide a comprehensive view of microbial diversity and functioning [7, 23–25]. In contrast, the use of advanced molecular techniques, such as PCR combined with environmental DNA (eDNA) metabarcoding, has optimized microbial ecology by enabling the simultaneous analysis of a broad range of microorganisms using high-throughput sequencing technology [26–34].

Environmental DNA (eDNA) metabarcoding enables researchers to identify and quantify microbial taxa through sequencing specific genetic markers from environmental samples, eliminating the need for microbial cultivation or isolation. This technique facilitates periodic sampling to monitor spatial and temporal shifts in microbial communities in response to environmental disturbances, making it a valuable tool for assessing pollution impacts in aquatic ecosystems [35, 36]. In riverine systems, eDNA metabarcoding has been employed to elucidate microbial community composition, structure, and dynamics along environmental gradients, revealing the effects of various stressors such as pollution, land use change, industrial activities, and human settlements [9, 17, 37–40]. This approach presents benefits but may introduce biases due to factors like DNA extraction efficiency, PCR amplification biases, and genetic marker selection. Successful implementations include the discovery of new microbial taxa in uncharted river systems and studies associating specific changes in microbial communities with pollutants. Nevertheless, despite growing eDNA metabarcoding use in river microbial community studies, there remains a significant lack of such research in tropical rivers [41–43].

The highly diverse geographic and climatic conditions in Mexico give rise to a wide array of ecosystems, including rivers that exhibit substantial gradients in conservation status and urbanization often leading to the degradation of river ecosystems [44]. While most studies on Mexican rivers have focused on water quality, pollutant concentrations, and macroinvertebrate diversity, few have explored the microbial communities within these systems, particularly using advanced molecular techniques such as rRNA gene amplicon sequencing. In this study, we employed rRNA gene amplicon sequencing, targeting the 16S and 18S rRNA genes to characterize prokaryotic and microeukaryotic communities across a pollution gradient in a tropical river within Mexico City. Moreover, we aim to identify key environmental factors influencing the structure of these communities and to gain insights into their composition in response to pollution-induced stress.

## Material and Methods

### Study Area

**Fig. 1:**
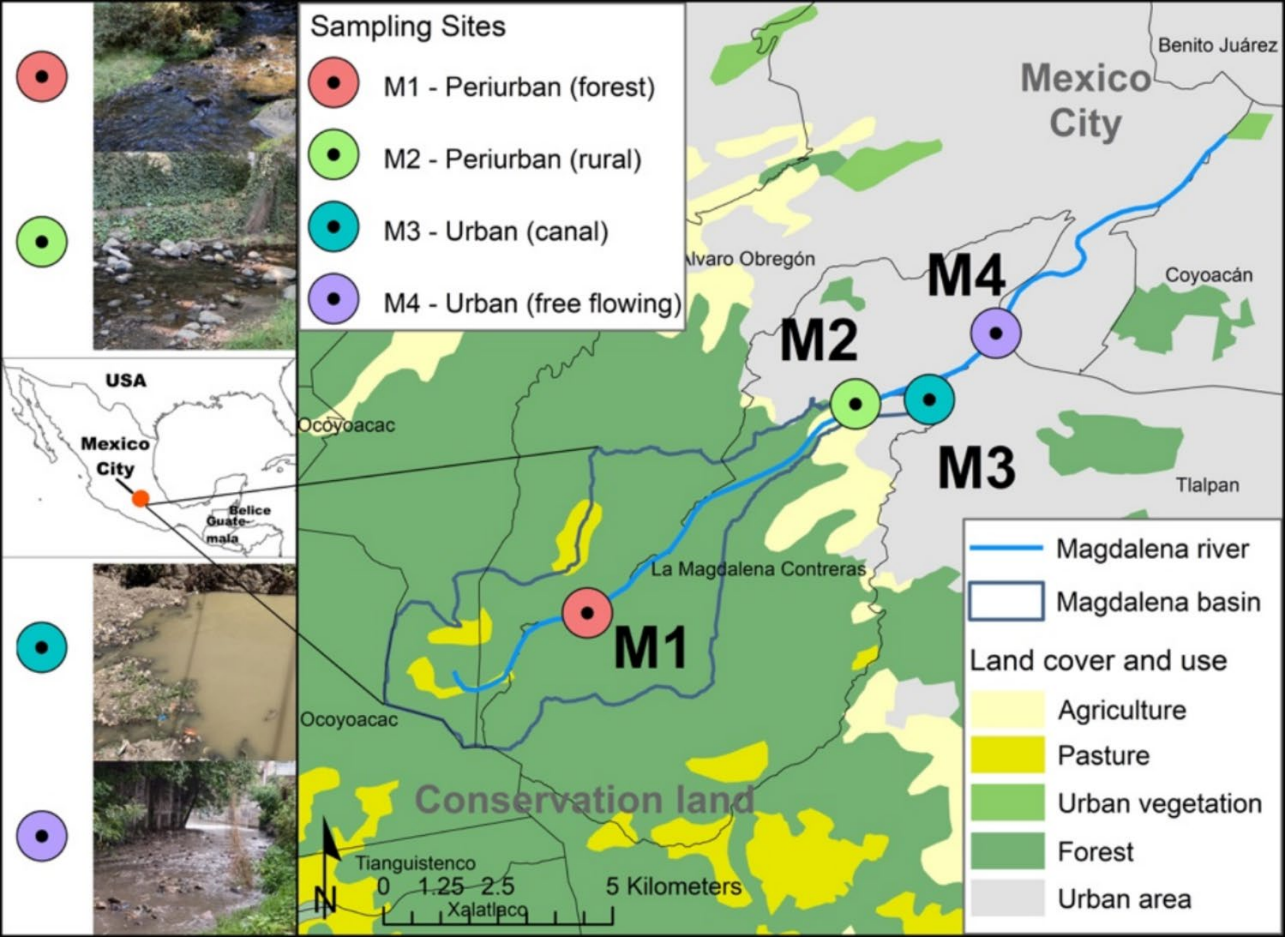
Location of the sampling sites along the Magdalena River in Mexico City

The Magdalena River, at 19° 15′ N, 99° 17′ 30″ O, begins in the Sierra de las Cruces at Mexico City’s southwestern edge, starting at 3800 m elevation. It flows 20 km into Mexico City’s valley at 2200 m. This river provides surface water for the city but receives untreated sewage from nearly 1,850,000 residents, becoming wastewater in urban areas [45]. The region has a tropical mountain climate, with annual rainfall of 2800 mm in the upper basin [46]. The Magdalena River is a vital freshwater ecosystem in Mexico City having distinct limnological features. Flowing from the southwestern mountains through urban zones, it affects water chemistry,also, the watershed’s seasonal precipitation influences flow rates and nutrient levels. In upper sections, water quality remains better, with higher dissolved oxygen and fewer pollutants. As it moves through populated areas, human impact increases, causing elevated organic matter, nutrients, and contaminants. The river’s pH ranges from slightly acidic to neutral, influenced by geology and human activities, and turbidity increases downstream from erosion and urban runoff (Jujnovsky 2012, [47]). We sampled four sites, covering a gradient of land uses, landscapes, and human influence (Table 1); the cumulative distance between the points is approximately 10 km; and this portion of the river is the part that remains exposed before it is piped further downstream. (Fig. 1; Table 1).

**Table 1:** Characteristics of the four sampling sites

Site	Location	Land cover	Landscape type	Human activities
M1	Upper basin	Pine-Oak forest	Periurban (forest)	Ecotourism
M2	Upper basin	Pine-Oak Forest and agricultural	Periurban (rural)	Tourism, agricultural and livestock
M3	Lower-basin	Urban area	Urban (canal)	Settlements and wastewater disposal
M4	Lower-basin	Urban area	Urban (free flowing)	Settlements and wastewater disposal

### Environmental Sampling for eDNA Extraction

**Fig. 2:**
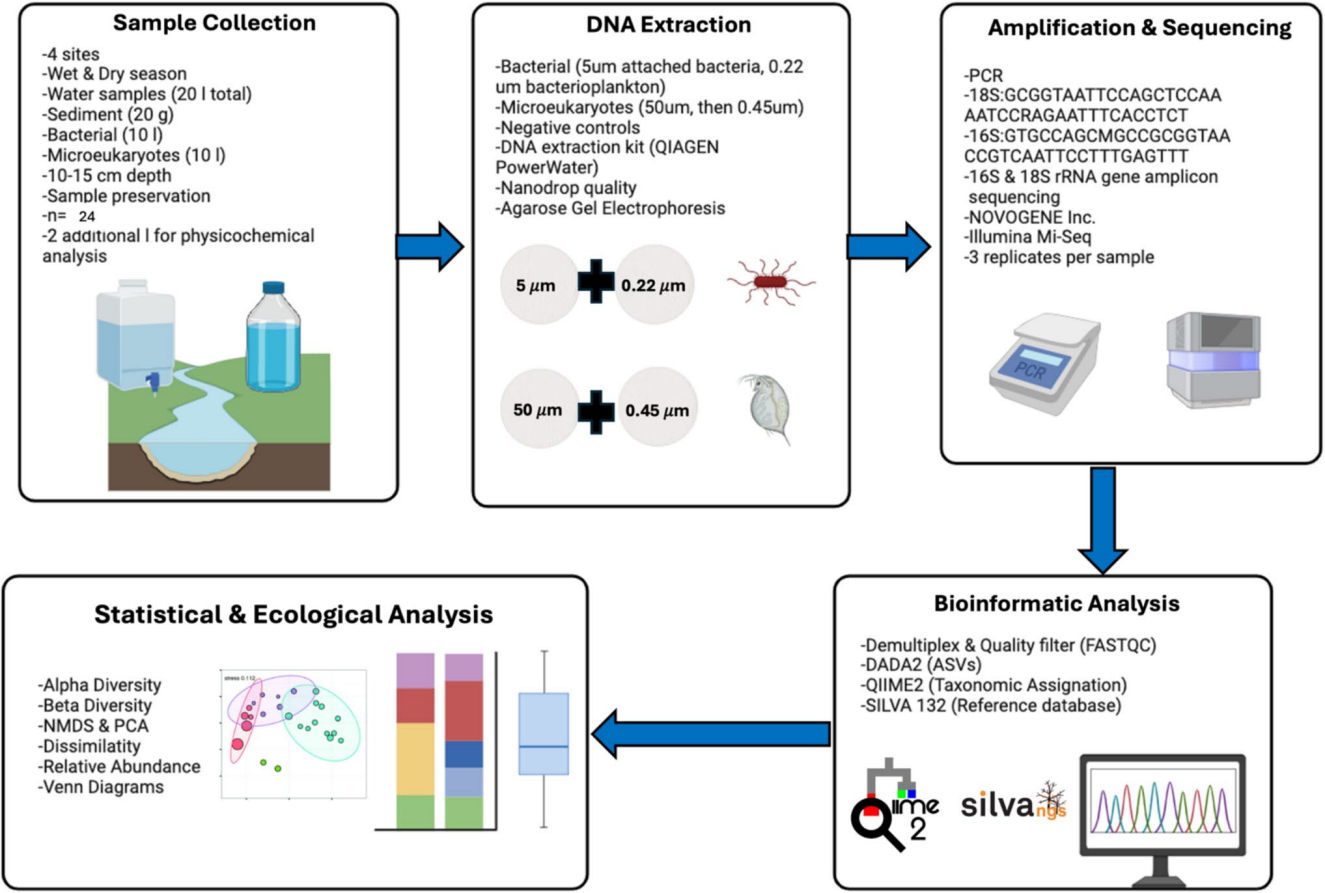
General workflow of field, laboratory and computational analysis steps for the present study

The four sites were sampled during the dry (February) and rainy (August) seasons of 2022. Water samples were collected using sterile plastic bottles in the accessible parts of the river, employing sterile gloves. For eDNA collection, the opening of the bottle was positioned 10–20 cm below the surface in areas of high-water flow. Also, for a mixed sample, collection of at least 20 g of sediment was done, as recommended by Cilleros et al. [48]. A 20-L container was pre-conditioned three times before being filled for subsequent sample processing in the laboratory (Fig. 2). This approach was adopted because working with samples that have larger volumes of water has been demonstrated to increase the quantity of eDNA collected in both laboratory-controlled environments and field conditions [49, 50]. Furthermore, for each sampling location, 1 L of distilled water was used as a negative control (blank, BL) from the laboratory, as well as two 1-L samples of river water.

### Physicochemical Characterization

In addition, 2 L of water were collected and transported to the laboratory (refrigerated in a cooler) for physicochemical analysis within 1 h of sample collection. Physicochemical properties such as nitrites (NO_2_), nitrates (NO_3-_), sulfides, and phosphates (PO_4_) were measured at the four sites using a HANNA multiparameter device, model DR900. Chemical oxygen demand (COD) was determined with the colorimetric method using HACH digester DRB200 and the DR900 portable colorimeter. For pH measurement, 200 mL samples were taken and analyzed with a HI5221-01 HANNA benchtop pH/mV meter with a research-grade accuracy of 0.001 pH. All samples were analyzed by triplicate according to protocol 2540 of the Standard Methods by the American Public Health Association (Table 2).

**Table 2:** Physicochemical variables for each sampling site in the dry and rainy season

Site	Season	Year	COD (mg/L)	NO_2_^−^ (mg/L)	NO_3_^−^ (mg/L)	PO_4_ (mg/L)	Sulfide (mg/L)	pH	T° (°C)
M1	Dry	2022	26.4	0.08	0.9	4.8	0.02	7.43	10
	Rain	2022	8.0	0.7	1.5	2.9	0.03	7.86	14.6
M2	Dry	2022	47	2.3	0.9	7.4	0.04	6.87	12.3
	Rain	2022	40	1.0	0.5	3.3	0.02	7.63	15.2
M3	Dry	2022	449	11.5	0.8	15	0.53	6.75	15.8
	Rain	2022	140	1.5	0.8	8.2	0.21	7.23	19
M4	Dry	2022	366.1	13.0	1.4	20	0.58	6.64	17
	Rain	2022	80	2.6	0.9	13.8	0.16	7.13	19.4

### DNA Extraction, Amplification, and Next-Generation Sequencing

To examine both free-floating and sediment-bound bacterial communities in the system under study, we applied the protocol suggested by Bairoliya et al. [51] and Brandao-Dias et al. [52], in which a differential vacuum filtration is employed to concentrate the two bacterial types. The process of isolating various eDNA particle sizes involved sequential filtration of each water sample (Fig. 2). Initially, for microeukaryotes and bacteria, all samples were passed through devices and filters with a 50-µm mesh opening to remove larger particles, particularly those associated with organism remains and plant matter. This was followed immediately by an additional filtration through 0.45 µm filters. For bacterial communities, the samples were then filtered through 5 µm mesh filters to capture bacteria attached to sediment-sized particles, and finally through 0.22 µm filters to collect bacterioplankton). Extracted DNA was then stored at − 80 °C for further analyses; a total number of 24 replicates considering triplicate for each site and season was processed. The environmental genomic DNA was extracted and obtained using the Dneasy Power Water Kit (Qiagen, Canada Inc.) as per the manufacturer’s instructions. The DNA quality was analyzed by agarose gel electrophoresis, and the absorption ratios of DNA at 260/280 nm and 260/230 nm were evaluated using an ND-2000 spectrophotometer (NanoDrop Inc., Wilmington, DE, USA). DNA fragments were amplified, and libraries were constructed with an average length of ~ 350 bp using the 150 paired-end sequencing strategy from the Illumina HiSeq platform. The sequencing was conducted by Novogene Bioinformatics Technology Co. Ltd. (Beijing, China). The gene for 16S and 18S ribosomal RNA was amplified using primers 515F: GTGCCAGCMGCCGCGGTAA and 907R: CCGTCAATTCCTTTGAGTTT for 16S and 528F: GCGGTAATTCCAGCTCCAA and 706R:AATCCRAGAATTTCACCTCT for 18S, respectively) targeting the V4–V5 hypervariable region with specific adapters and barcodes for multiplexing (BioProject ID PRJNA1171456).

### Bioinformatic Analysis

The computer analysis was performed using the bioinformatic cluster of the Instituto de Biotecnologia-UNAM. QIIME2 [53] was employed to execute several key tasks: The DADA2 plugin was utilized for sequence quality control and the creation of a feature table, which included the correction of amplicon sequence data, the removal of phiX reads and chimeric sequences, and the excision of primer sequence nucleotides. Following this, forward and reverse sequences were trimmed to a maximum length of 250 nucleotides. The sequences that remained were grouped into amplicon sequence variants (ASVs), which facilitated denoising and provided fine-scale, capable of resolving differences down to a single nucleotide [54–56]. Sequenced samples underwent rarefaction to achieve consistent sequencing depths: a. 100,000 bp for 18S b. 90,000 bp for 16S. VSEARCH was used to detect and remove chimeric sequences. We chose to use ASVs to represent exact sequence variants because they offer several benefits over traditional OTU clustering [54, 56] (Figure S1).

### Statistical Analysis

The R software was utilized to calculate alpha diversity indices, including Sobs, Chao1, ACE, and Shannon indices, through the use of the “phyloseq” and “vegan” packages [57, 58]. The “phyloseq” package was employed to analyze the structure and composition of microbial communities. To evaluate beta diversity, principal component analysis (PCA) and non-metric multidimensional scaling (NMDS) were performed. Furthermore, the influence of physicochemical variables on microbial community composition was assessed using permutational multivariate analysis of variance (PERMANOVA) and analysis of similarities (ANOSIM), both based on Bray–Curtis dissimilarity matrices computed with the adonis function from the “vegan” package. These multivariate approaches enabled a comprehensive evaluation of community structure in relation to environmental gradients.

## Results and Discussion

### Changes in Physicochemical Environmental Variables as Determinants for Microbial Community Composition

The physicochemical characterization data indicate that concentrations of NO₃^−^, NO₂^−^, PO₄^3−^, and COD progressively increase from M1 to M4, corresponding with increasing levels of human influence—a pattern that aligns with observations for most rivers, including tropical ones [8, 59–63] (Table 2). This pattern is consistent across both seasons. Notably, nitrite levels exceeded the permissible limits set by Mexican water quality regulations at all four sites [4]. High nitrite concentrations are generally associated with nitrite oxidation and may indicate contamination from fecal matter or agricultural fertilizers. The remaining parameters, however, did not exceed the Mexico’s established limits.

**Fig. 3:**
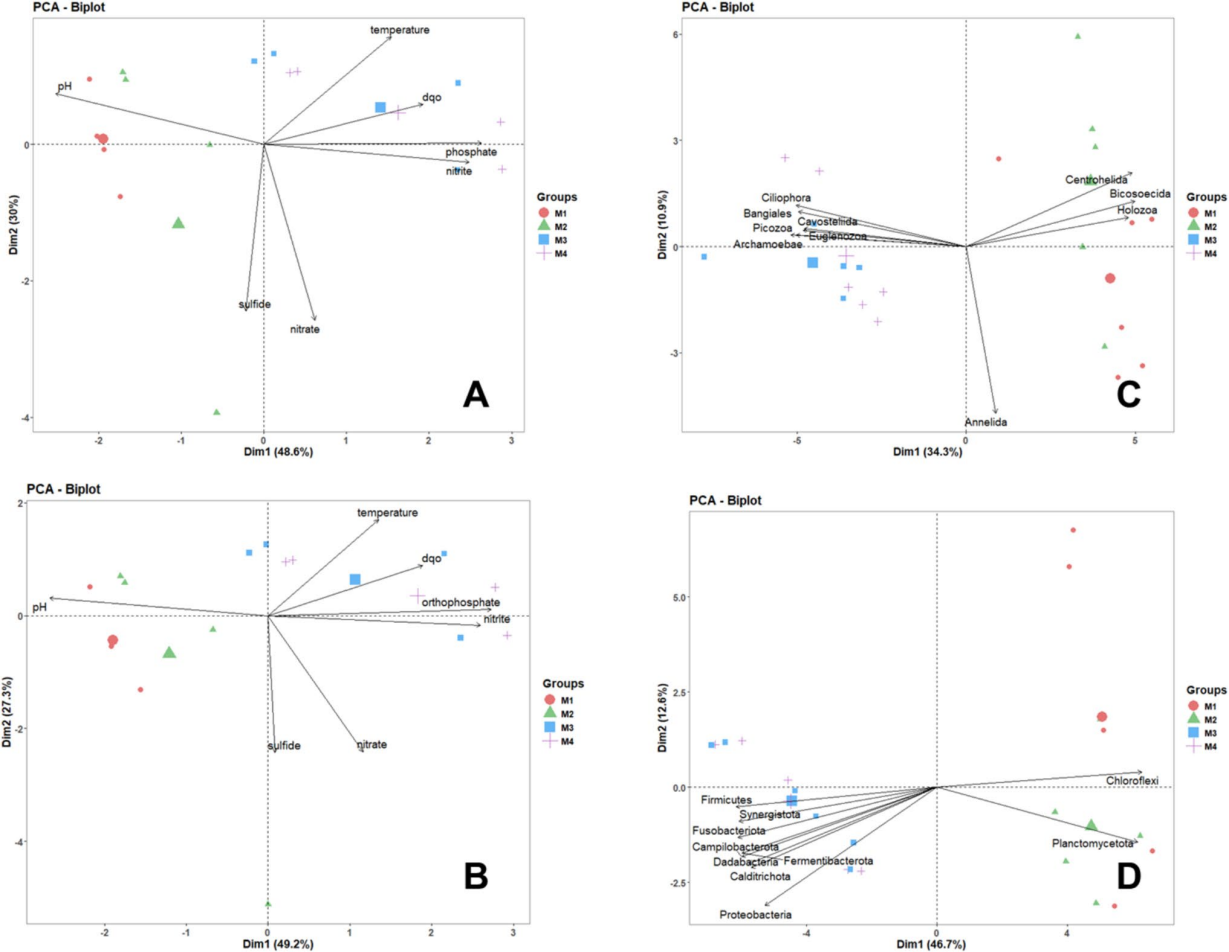
Principal component analysis (PCA) biplots where **A** and **B** explain the water quality variation in chemical factors respect to sites and **C** and **D** represent the correlation of sites with microbial groups at phylum level for **C** microeukaryotes and **D** prokaryota

The principal component analysis (PCA) of physicochemical variables shows differences between urban and peri-urban sites (Fig. 3). For both microeukaryotic and prokaryotic communities at the phylum level, the first component relates to COD and the second to PO₄^3−^. In the case of beta diversity, the first two components explained 76.5% of total variance (49.5% and 27.28%, respectively) for prokaryotic communities, while for eukaryotic communities, 59.3% of total variance is explained (46.7% and 12.5%, respectively) (Table S5; Fig. 3). Regarding sites M3 and M4, the principal components are related to COD, phosphorus, and sulfide, while sites M1 and M2 do not show a clear relationship to assessed environmental parameters (Fig. 3).

The PCA for ASVs shows that changes in physicochemical properties affect the microeukaryotic, as well as prokaryotic communities. This aligns with research indicating that sewage can enhance microbial communities with species that may be pathogenic [59–62, 64, 65],in this scenario, bacterial groups are primarily composed of Firmicutes, Campylobacterota, and Fusobacterota, all of them containing some of the most common and recognized genus as pathogenic [64–69].

**Fig. 4:**
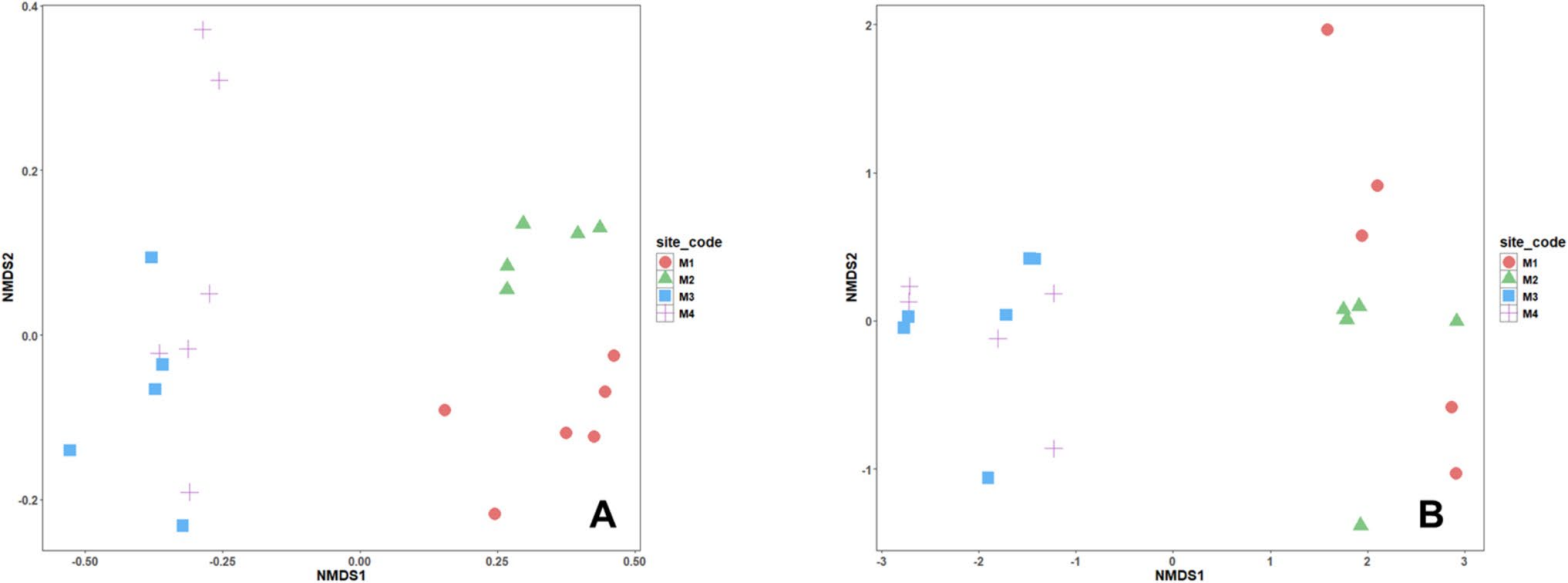
Non-metric multidimensional scaling (NMDS) of community composition based on Jaccard dissimilarity, where **A** and **B** explain the similarity between sample sites and **C** and **D** explain the grouping of those sites with microorganisms, for **A**–**C** phylum and **B**–**D** genus

While temporality has no obvious effects on beta diversity, NMDS analyses indicate for microeukaryotic and prokaryotic communities that the ordination distinguished two groups: the first one, composed by M3/M4, and the second one, by M1/M2, without overlapping of replicates from different sites (Fig. 4) (ANOSIM statistics *p* < 0.01, *R* = 0.84 and *p* < 0.01, *R* = 0.86 for phylum level, and *p* < 0.01, *R* = 0.79 and *p* < 0.01, *R* = 0.71 for genus level).

A total of 3,294,644 high-quality (with a Phred score > Q20) reads were obtained by high-throughput sequencing of 16 samples from the four sites. The average number of reads per sample was 106,539.09 ± 9,085 (mean ± SE) for the 16S-V4 amplicon and 114,031.04 ± 6,444 (mean ± SE) for the 18S-V4 amplicon. A total of 1,171,930 prokaryotic (16S-V4) and 2,122,714 eukaryotic reads (18S-V4) were identified. The saturation of the rarefaction curves (Figure S1) indicate that the sequencing data could be used for a posterior analysis. Metabarcoding of prokaryotic organisms showed that the phyla with the greatest abundances were Proteobacteria (45%), followed by Cyanobacteria (16%), Campylobacterota (14%), Bacteroidota (11%), and Verrucomicrobiota (2%) (Fig. 4; Figure S2-A; Table S1); in the 16S dataset, 626 ASVs were assigned to genus, 627 to family, 148 to order, and 54 to phylum. For eukaryotic organisms, the analysis revealed 61 phyla. The phyla with the greatest mean abundances were Ciliphora, (31%), followed by Chlorophyta (22%), Diatomea (13%), Phragmoplastophyta (6%) Rotifera (6%), and Arthropoda (4%; Figure S2-B; Table S2); in this 18S dataset, 227 ASVs were assigned to Genus, 116 to Family and 117 to Order.

### Changes in Prokaryotic Communities

**Fig. 5:**
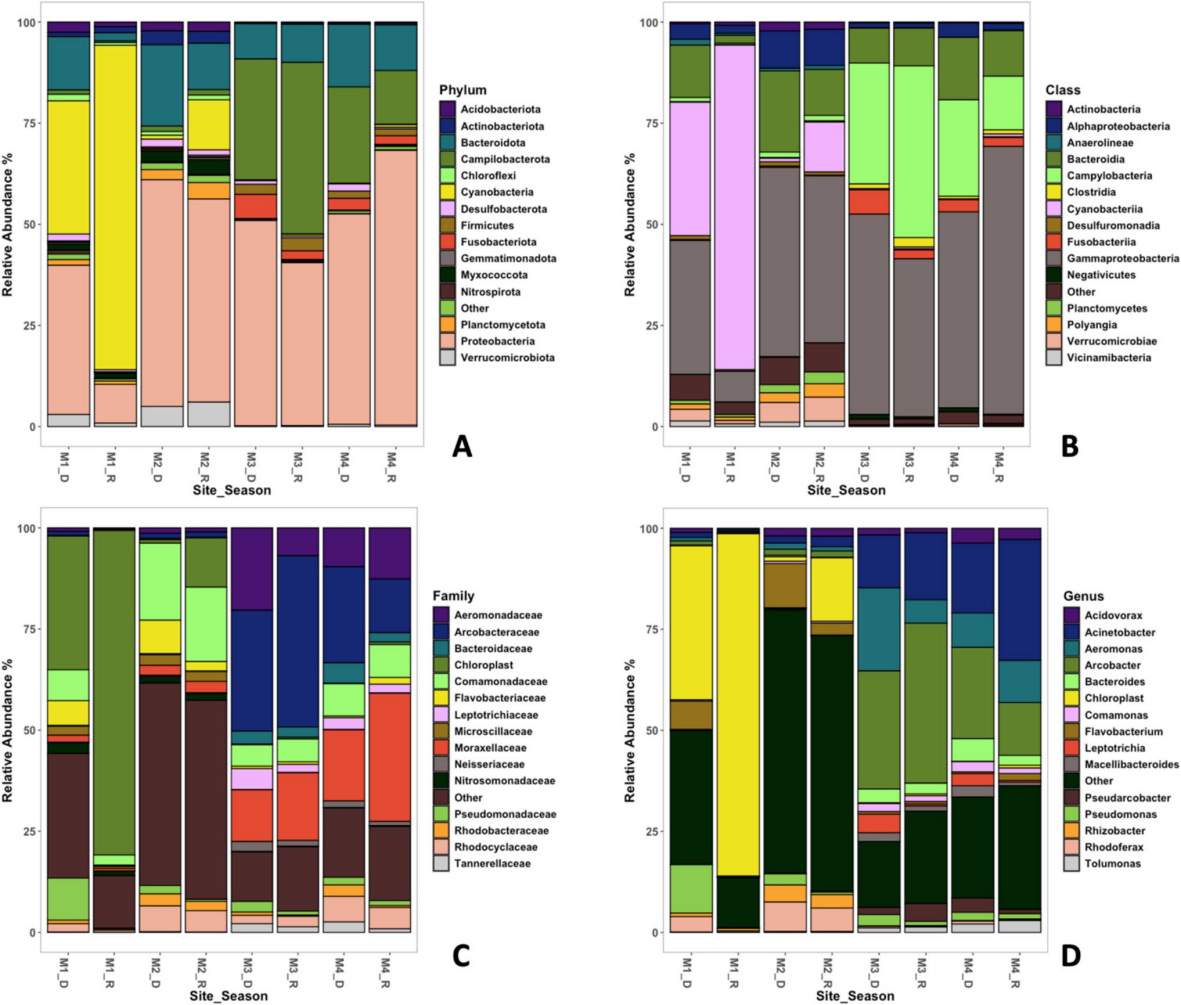
Composition for prokaryotic communities in relative abundance, for all sites and seasons (2022) at the Magdalena River (Mexico City). Characterization was made using the top 15 groups and considered the taxonomic levels of phylum (**A**), class (**B**), family (**C**), and genus (**D**)

Distinct distribution patterns of prokaryotic community composition are observed among sampling sites (Fig. 5). The dominant phylum at site M1 is Cyanobacteria (54%), while for M2, M3, and M4, Proteobacteria is dominant; in this study, it was noted that Alphaproteobacteria were not present in significant relative abundances among Proteobacteria. However, for site M1, there was a significant difference for Gammaproteobacteria (*p* < 0.05) (Fig. 5B). The relative abundance of Cyanobacteria declines with increasing pollution, so that although present at M2, M3, and M4, Cyanobacteria only represent 6%, 0.3%, and 0.2%, respectively. Other phyla with the same pattern of decreasing abundance with increasing pollution are Acidobacteriota, Nitrospirota, Planctomycetota, and Verrucomicrobiota (Fig. 5A). Phyla with an inverted pattern of being dominant at urban sites (M3 and M4) are Campylobacterota, Bacteroidota, Firmicutes, and Fusobacteriota, the latter two being virtually absent at sites M1 and M2 (0.1% and 0.1% for M1; 0.3% and 0.2% for M2, respectively). Furthermore, these two last phyla are associated with genera of intestinal microbiota, as well as domestic sewage discharges [66–68, 70].

Temporal differences are detected in the presence, as in the abundance of prokaryotic groups (Cyanobacteria increases and Bacteroidota decreases in rainy season) along the pollution gradient in the river (Fig. 5), which could be related to the dilution effect of strong and sustained precipitation on rainy season. This could suggest an influence of the seasonal component, represented by the sampling date.

The dominant groups of peri-urban sites (M1 and M2) are the phyla Cyanobacteria, Planctomycetota, Myxococcota, and Verrucomicrobiota. The presence of Cyanobacteria in high relative abundances is often associated with risks due to cyanotoxin formation [71, 72]; however, other studies refer to this group as forming the indigenous bacterial communities of rivers [11]. The phyla that are present in all sites and seasons are Proteobacteria, Bacteroidota, and Actinobacteria, that is consistent with vertebrates’ microbiota (Fig. 5A). Planctomycetota and Myxococcota were mainly found in sites with rural land use (M2), and these groups are often considered taxa with saprophytic habits and key players in microbial interactions, often associated with biofilms, algae, cyanobacteria, and usually accompanying the phylum Verrucomicrobiota (Fig. 5A, [73]). Additionally, some studies have associated the presence of the phylum Verrucomicrobiota with the onset of urbanization [70, 73]. This study confirms this finding, as the abundances at the most contaminated sites (M3 and M4, with 0.8 and 1% respectively) were lower, suggesting that the group could be a potential indicator of human influence from a periurban perspective.

Proteobacteria and Bacteroidota together dominate the bacterial communities in all sites and seasons (around 60%), except for M1 in the rainy season (Table S1). These results are consistent with studies that report Proteobacteria, Bacteroidota, and Actinobacteria changing their relative abundance depending on the degree of human influence [33, 59, 68, 74]. Specifically, in M1, there were low levels of beta and gamma bacteria, including genera like *Rhizobacter* and *Rhodoferax*, which are typically linked to environments with low to moderate contamination [75–77]. In M2, Betaproteobacteria showed the highest relative abundance, as seen in the levels of *Rhizobacter* and *Rhodoferax*. Betaproteobacteria are generally more prevalent in freshwater systems and can thrive in diverse environmental conditions. However, their abundance tends to rise with increasing pollution, especially in areas with high ammonia nitrogen and total nitrogen levels [64, 65, 75–77]. Thus, in M3 and M4, which showed high nitrogen levels, there were high relative abundances of both Betaproteobacteria and Gammaproteobacteria, including genera such as *Acinetobacter*, *Aeromonas*, *Comamonas*, *Tolumonas*, and *Acidovorax*. It is important to note that in general, Gammaproteobacteria show similar patterns to Betaproteobacteria in response to pollution, with increased abundance in more polluted waters [75, 78]. They are often associated with sulfur cycling in anaerobic conditions, particularly in marine sediments [64, 65, 79]. Thus, the distribution and abundance of various Proteobacteria classes in river systems are significantly affected by pollution levels, nutrient concentrations, and seasonal changes, and their presence and relative abundances can serve as indicators of water quality and ecosystem health in freshwater environments.

Similarly, the phyla Bacteroidota and Actinobacteria are well known for their involvement in organic matter decomposition in sites with high concentrations of organic matter [11, 80, 81]. Due to their eco-physiological characteristics, these phyla can survive and even proliferate in environments associated with wastewater or domestic water discharges [59, 70, 82]. Consequently, their presence is often indicative of elevated anthropogenic activities and may signal the potential occurrence of antibiotic resistance genes [37, 67, 83]. However, their relative abundances do not change along the gradient of human influence (Table S1), contrary to what has been documented by several other studies [37, 67, 83].

A characteristic feature of urban sites (M3 and M4) is the presence of the phyla Campylobacterota, Fusobacterota, and Firmicutes. Firstly, the presence of Campylobacterota in high abundances is often used as indicators of anoxic conditions in water with elevated sulfur content, especially for bacteria belonging to the genera *Sulfuromales* and *Arcobacter* [69, 73, 81], as observed in the urban sites. Secondly, Firmicutes, as a major phylum of bacteria commonly found in the human gut microbiome, can indeed serve as a valuable bioindicator of water quality, particularly in relation to fecal contamination in rivers. Their presence in high densities can indicate contamination from sewage or other fecal sources, making them effective bioindicators of water pollution, as stated by many studies [69, 70, 73]. Therefore, a high relative abundance, or even dominance, is considered an important indicator of fecal contamination [66, 70, 82, 84, 85] *Bacillus*, *Staphylococcus*, and *Clostridium*, all belonging to Firmicutes [66, 84, 85]. Although in this study, they were not among the most abundant genera at any sampling point or season, but it is notorious that their relative abundance was evident in urban sites (Fig. 5D).

Similar patterns among peri-urban and urban sites are also evident at the Class and Family taxonomic levels as reported in several studies (Fig. 5 B and C, respectively). Those studies stated that in peri-urban areas, classes such as Polyangia, Vicinibacteria, Verrucomicrobiae, and Planctomycetes, along with families like Flavobacteriaceae, Microscillaceae, and Nitrosomonadaceae, are characteristic. In contrast, urban sites (M3 and M4) display higher relative abundances of classes such as Campylobacteria, Fusobacteriia, and Clostridia, as well as families including Aeromonadaceae, Arcobacteraceae, Bacteroidaceae, Leptotrichiaceae, Moraxellaceae, Neisseriaceae, and Tannerelaceae. (Fig. 5C) [69, 70, 73, 82].

Finally, the analysis at the finest taxonomic level in this study demonstrates that some of the genera present along the human influence gradient can be used as potential bioindicators, as they exhibit specific eco-physiological characteristics that determine their presence or absence under certain conditions. At the genus level, the analysis of prokaryotic communities shows a pattern consistent with the previously described phyla, demonstrating a particular composition of communities in peri-urban and urban sites (Fig. 5D). Such is the case of peri-urban sites where genera like *Flavobacterium*, *Rhizobacter*, and *Rhodoferax* are present in significant abundances. These genera are usually involved in the nitrogen cycle, recycling or making this element available, or they are involved in plant–microbe interactions, so the presence of these genera in these sites can be an indicator of medium to low anthropogenic influence derivates from agricultural activities [69, 73].

The urban sites M3 and M4 share similar relative abundances and dominance patterns, which differ from those observed in M1 and M2. In urban areas, phyla Proteobacteria, and Campylobacteria, with some of their genera such as *Acinetobacter*, *Aeromonas*, *Arcobacter*, *Pseudomonas*, *Pseudoarcobacter*, *Leptotrichia*, and *Tolumonas*, are dominant [76, 77]. These genera are known for being pathogenic, often of nosocomial origin or associated with mammalian enteric systems. Their persistence in highly contaminated environments characterized by high organic matter content and low oxygen levels is also linked to a high prevalence of antibiotic resistance genes [66, 70, 84–86]*.*

### Changes in Microeukaryotic Communities

**Fig. 6:**
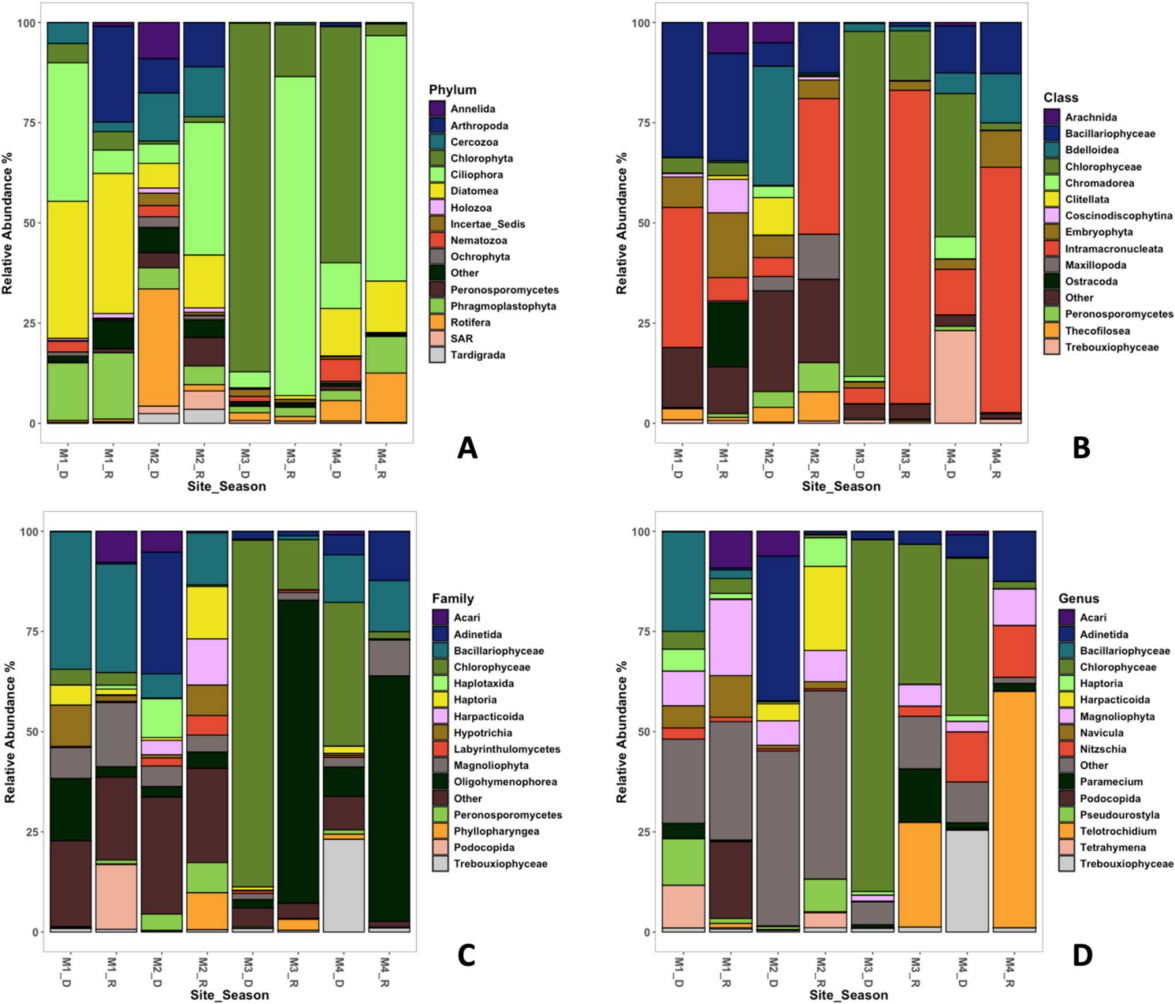
Composition for microeukaryotic communities in relative abundance, for all sites and seasons (2022) in Magdalena River, Mexico City. Characterization was made using the top 15 groups for phylum (**A**), class (**B**), family (**C**), and genus (**D**) levels

In the case of microeukaryotes, it was possible to identify differences between sites in terms of community diversity, as well as relative abundances at all four taxonomic levels (Fig. 6). The dominant phylum at the forest site (M1) is Diatomea (34%), while for intermediate to strongly polluted sites (M2, M3, M4), Ciliophora is the dominant phylum (18%, 42%, 39%, respectively; Fig. 5A). The relative abundance of Diatomea declines with increasing pollution, so that although present at M2, shows lower abundance (9%); for M3 and M4, Diatomea only represent 0.5% and 11%, respectively. Other phyla with the same pattern of decreasing abundance with increasing pollution are Annelida, Arthropoda, Cercozoa, Ochrophyta, and Tardigrada (Fig. 6A). Phyla with an inverted pattern of dominance at urban sites (M3 and M4) but with decreasing abundance in the upper watershed (M1 and M2) are Chlorophyta, Ciliophora, and Rotifera. For urban sites (M3 and M4), Chlorophyta and Ciliophora presented pronounced changes in their contributions at the different seasons (Fig. 6A). Diatoms, Cercozoa, Tardigrades, and Arthropods, could be considered as indicators of low to medium human influence levels. as their functional role is as producers and members of intermediate trophic levels [87–91]. On the other hand, a reduction in eukaryotic phyla and dominance by a few, such as Ciliates in urban sites, are associated with high contamination or human influence, leading to a reduction in biodiversity associated with environmental degradation processes [86, 92, 93].

Across all samples and seasons along the gradient, Ciliophora was consistently present, accounting for an overall dominance of 31%. Additionally, Chlorophyta and Diatomea together comprised approximately 53% of the eukaryotic communities. (Table S2). High concentrations of Ciliophora and Chlorophyta often indicate nutrient-rich environments caused by organic contamination [94]. Moreover, ciliates thrive in low-oxygen conditions, thus, suggesting possible hypoxia [95, 96]. These conditions may suggest organic pollution or nutrient enrichment for all of the study area. On the other hand, a particular case is that for the composition of microeukaryotic communities at M2, that differs from that of urban sites as it can also be observed in prokaryotic communities (Fig. 6).

These same patterns between peri-urban and urban sites can also be observed in taxonomic analyses at the class and family levels (Fig. 6 B and C, respectively), showing classes and families characteristic of peri-urban areas (M1 and M2), such as Bacillariophyceae, Clitellata, and Thecofilosea, as well as families Acari, Haplotaxida, Haptoria, Harpacticoida, Hypotrichia, and Peronosporomycetes. Conversely, classes and families characteristic of urban sites (M3 and M4) include Chlorophyceae, Intramacronucleata, and Trebouxiophyceae at the class level and Chlorophyceae Oligohymenophoreae at the family level (Fig. 6C).

At the genus level, the analysis of microeukaryotic communities shows a pattern consistent with the previously described phyla (Diatomea and Ciliophora), demonstrating a particular composition of communities in peri-urban and urban sites (Fig. 6D). At site M1, there are high abundances of *Navicula*, *Pseudourostyla*, and *Tetrahymena.* For site M2, the community composition is distinct, with *Adinetida* and *Harpacticoida* being dominant. In contrast, urban sites M3 and M4 exhibit similar relative abundances and dominance patterns that differ from those in M1 and M2, with genera such as *Nitzchia*, *Telotrochidium*, and *Paramecium* prevailing in urban areas (Fig. 6D).

Two diatom genera, *Navicula* and *Nitzschia*, were present in periurban locations with a combined average relative abundance of 6%. In urban areas, *Navicula’s* relative abundance was below 1%, while *Nitzschia* averaged 8%. *Nitzschia* typically exhibits a broader ecophysiological tolerance than *Navicula*, suggesting that urban conditions may be unfavorable for *Navicula*. Those data are consistent with [97] and [98]. Palmer [97] identified *Nitzschia* as one of the top five pollution-tolerant genus. Salomoni et al. [98] placed *Nitzschia* in a group, encompassing species more resilient to severe organic pollution and eutrophication. The same research placed *Navicula* in a category of tolerant but more widely distributed species, implying lower pollution tolerance than *Nitzschia* [98]. In summary, although both *Nitzschia* and *Navicula* demonstrate pollution tolerance, the cited studies indicate that particularly *Nitzschia* exhibits a higher pollution tolerance range compared to *Navicula* species, supporting the findings in this study.

Several studies employing 18S amplicons report that the predominant groups of eukaryotes in rivers are Holozoans (Animals) and Chlorophytes (green algae). Within microeukaryotes, it has been documented that the following taxa dominate: Apicomplexa, Arthropoda, Ascomycota, Basidiomycota, Chlorophyta, Cnidaria, Bacillariophyta, Euglenophyceae, Glomeromycota, Nematoda, Platyhelminthes, and Streptophyta [87, 99, 100], which coincide in part of our results.

### Shared Amplicon Sequence Variants Between Sites and Seasons

**Fig. 7:**
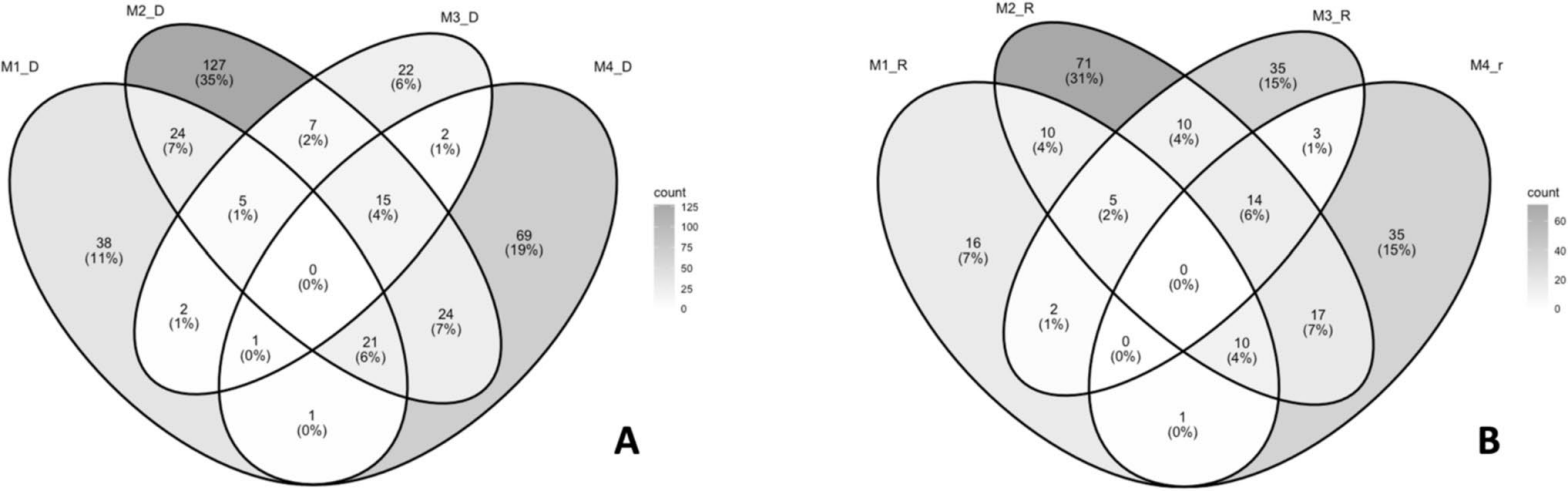
Venn diagrams showing shared ASVs between sites and seasons. **A**–**B** Bacterial communities for dry and rain seasons, respectively

**Fig. 8:**
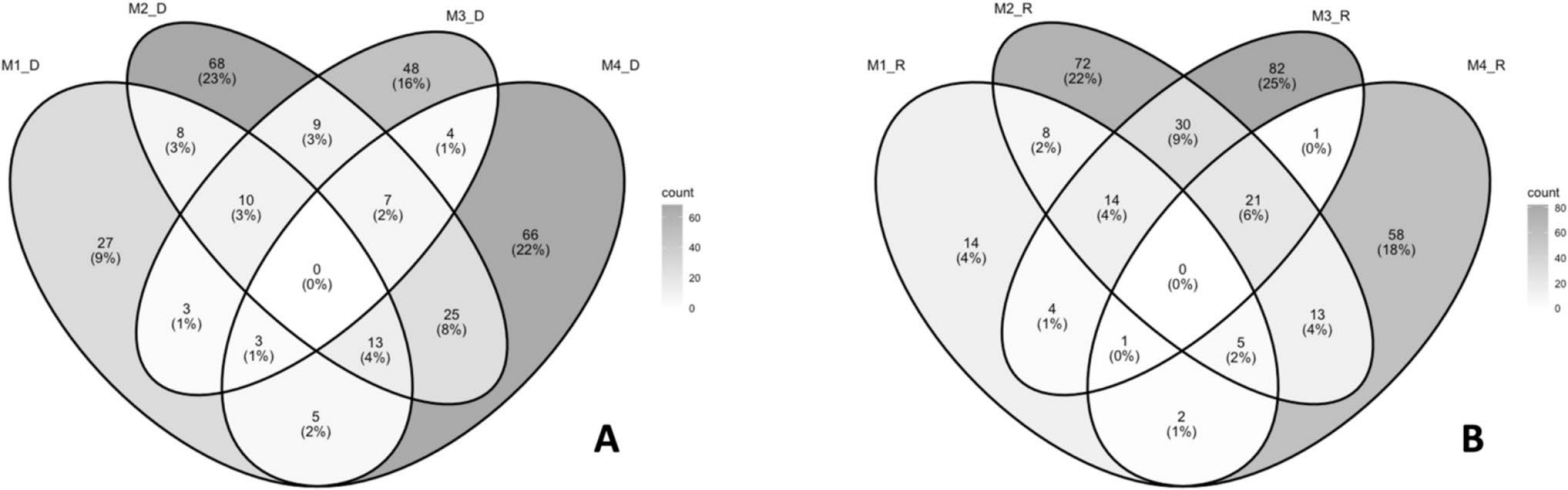
Venn diagrams showing shared ASVs between sites and seasons. **A**–**B** Eukaryotic communities for dry and rain seasons, respectively

Overall, for both organism groups and across time, similar spatial and temporal patterns of ASVs were observed: Urban sites shared a higher number of ASVs, while M1 exhibited the fewest ASVs in common with urban areas. In contrast, M2 shared many ASVs with the other three sites but also possessed a greater number of unique ASVs (Figs. 7 and 8).

Among the calculated diversity indices, both seasons and organism groups, the peri-urban site M2 exhibits the highest diversity, followed by the forested site M1. Conversely, urban sites (M3 and M4) show a lower diversity (Figures S-3, S-4).

## Conclusions

This research provides the first characterization of microbial communities in rivers of Mexico City, as well as their alterations as they pass through human-impacted environments. Moreover, the observed compositions of both microeukaryotic and bacterial communities generally corroborate findings from numerous previous studies in temperate rivers. However, this research identified certain taxa that were not previously documented in the extant literature, which predominantly focused on rivers in temperate regions. The results show that the phyla Firmicutes, Fusobacteriota, and Campylobacterota represent potential bioindicators of high anthropogenic influence, as has been reported for rivers of temperate latitudes under human influence. Our results suggest that phyla like Cyanobacteria, Chloroflexi, Verrucomicrobiota, Myxococcota, and Planctomycetota could be potential bioindicators of low levels of human activities and environmental alterations typical for peri-urban areas where urbanization is encroaching into rural areas.

In the case of bacteria, enteric, nosocomial, or fecal bacteria are indicative of medium to high disturbance (*Acinetobacter*, *Aeromonas*, *Arcobacter*, *Pseudomonas*, *Pseudoarcobacter*, *Leptotrichia*, and *Tolumonas*), while those associated with nitrogen biogeochemical processes or involved in plant–microbe interactions are indicators of low to medium disturbance (*Rhodoferax*, *Flavobacterium*, *Rhizobacter*).

For microeukaryotes, a greater number of phyla can indicate a better condition of the ecosystem, as the presence of groups occupying intermediate and top trophic levels (arthropods, tardigrades, cercozoa) indicates a better flow through trophic levels.

Some genera of diatoms, such as *Nitzchia* and *Navicula*, and ciliates (*Telotrochidium*, *Paramecium*) are routinely used to assess the level of contamination in rivers. The primary advantage of our study lies in the disaggregation of relative abundances across various taxonomic levels, enabling a comprehensive examination of the most appropriate taxonomic level for elucidating compositional changes. Thus, we consider that a combination and linkage between phyla and genera of microbial communities, can give a robust determination. The later can be proposed because using the phylum taxonomic level can provide a good representation of the pressures on the system and the groups that can persist under them, depending on their ecophysiological tolerance thresholds; while analysis at the genus level helps elucidate the dynamics, interactions, and long-term responses to these conditions. Finally, reaching the finest possible taxonomic resolution (species) could be a very powerful tool regarding the specific risks associated with changes in microbial community composition, however, to achieve this level, it is necessary to increase available genomic datasets from aquatic systems. Furthermore, the identification and confirmation of species through conventional ecological studies will be a cornerstone of community change characterization.

## Supplementary Information

Below is the link to the electronic supplementary material.

ESM 1 (1.30 MB DOCX)

## Data Availability

Raw sequencing data were deposited in the NCBI Bioproject database (BioProject ID PRJNA1171456).
