# Supplementary material for: Changes in Microbial Community Assemblages Due To Urban Pollution, Detected via rRNA Gene Amplicon Sequencing in the Magdalena River, Mexico City
Source: Microb Ecol. 2025 Aug 2;88(1):85. doi: 10.1007/s00248-025-02580-7 (PMC12317890; doi:10.1007/s00248-025-02580-7)
Supplement: Supplementary file 1 — (1.30 MB DOCX) [file 248_2025_2580_MOESM1_ESM.docx]

**Supplementary Material**

**
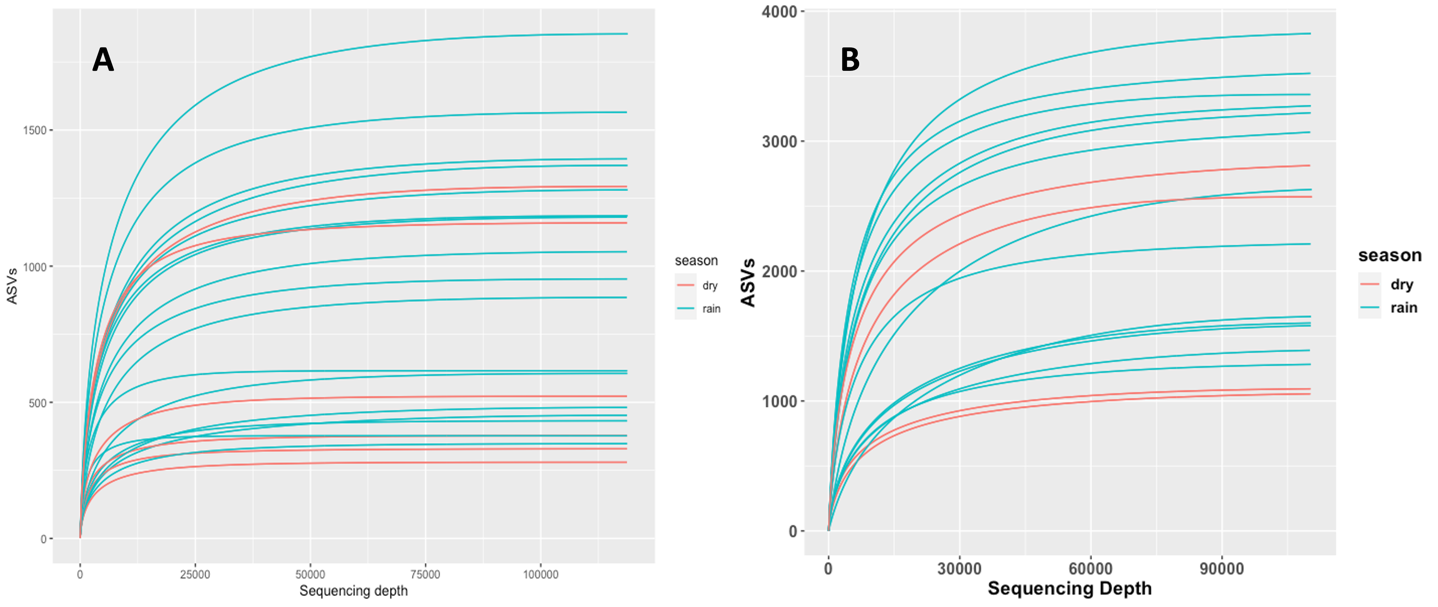
**

Figure S1. Rarefaction curves for all the samples used in this study and their respective seasons. A) 18S-V4 Eukarya samples n=23; B) 16S-V4, V5 Bacterial Samples.


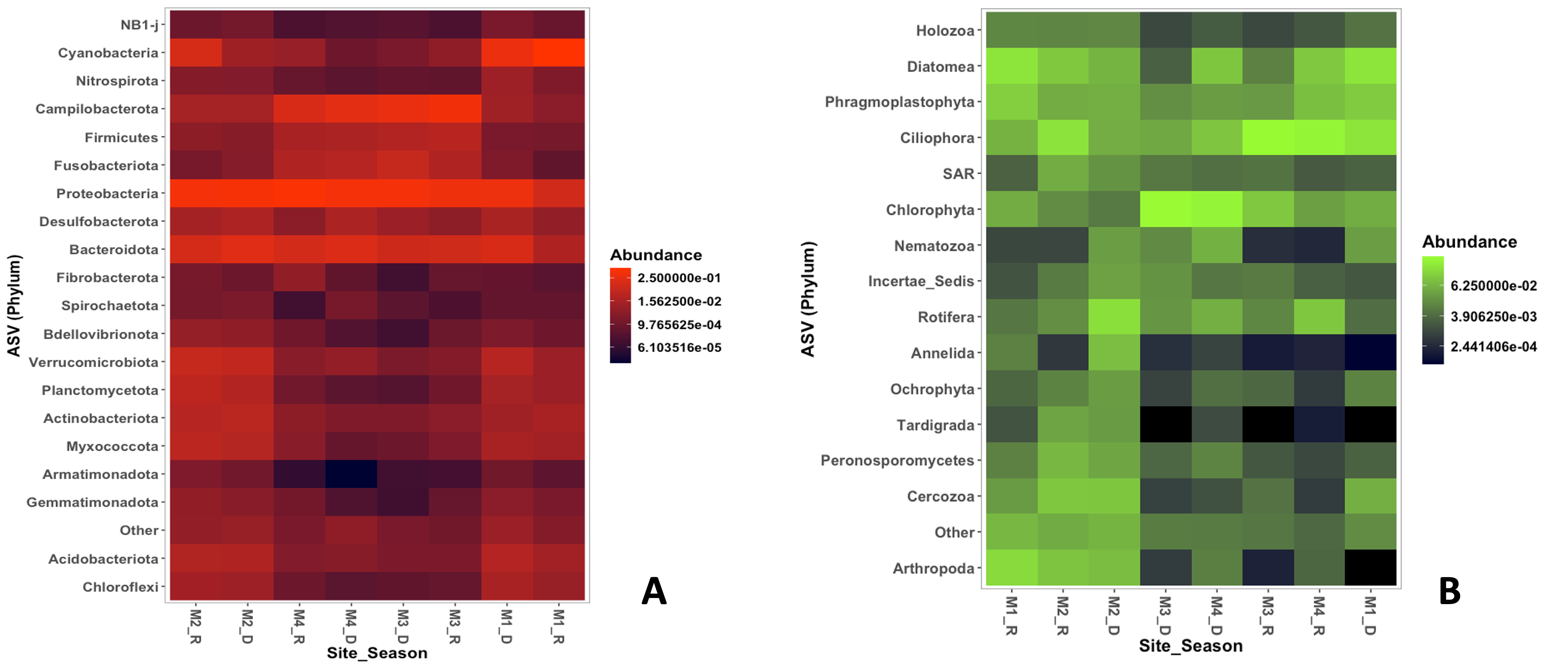


Figure S2. Heatmaps showing the top 20 and top 15 average abundant phyla for the samples in Magdalena River. A) 16S Bacterial B) 18S Eukarya.

Figure S-3. Diversity Indexes based on ASVs (Shannon, Simpson, InvSimpson) applied to Prokaryotic communities as well as average diversity for each site.


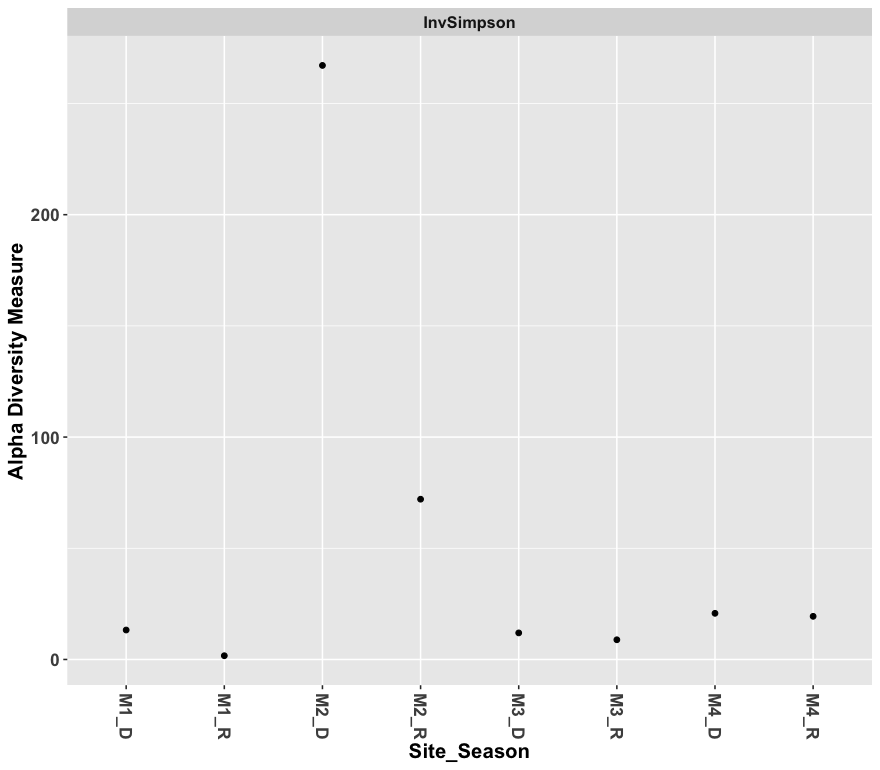

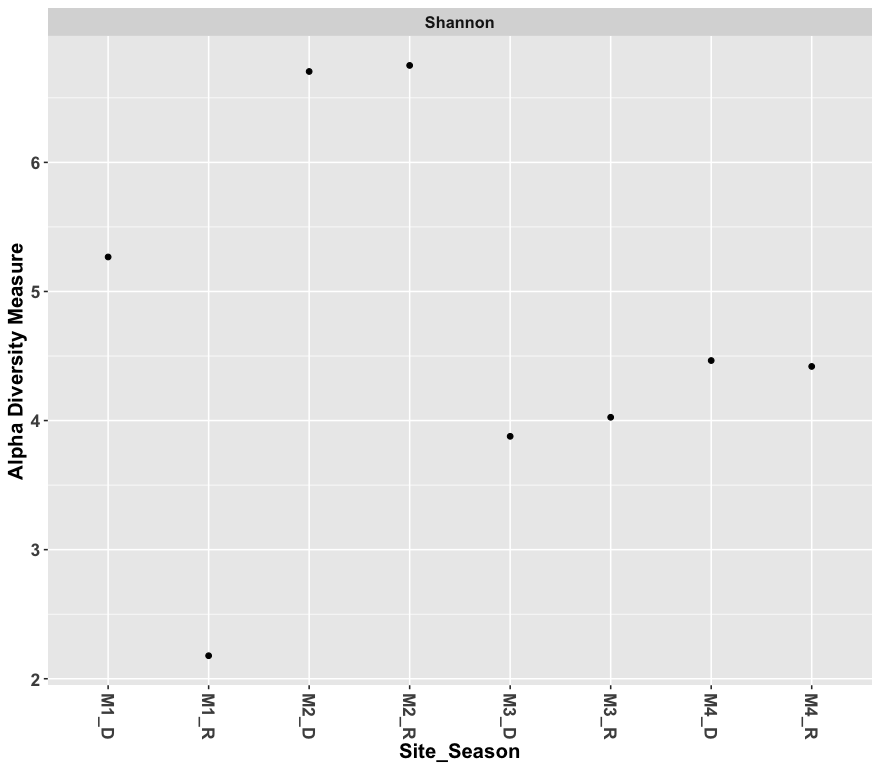

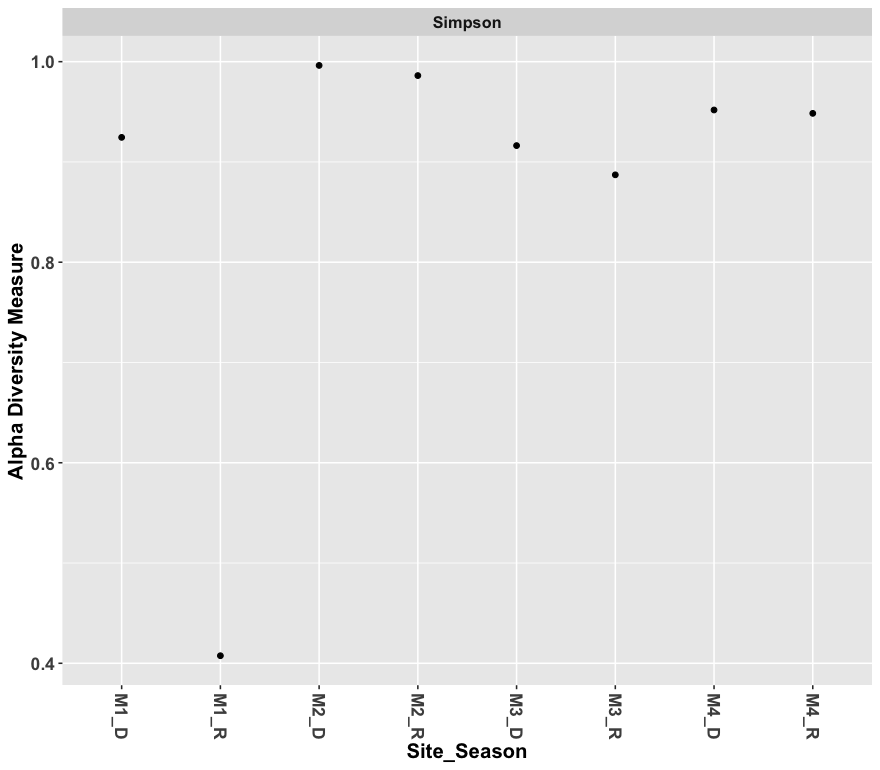


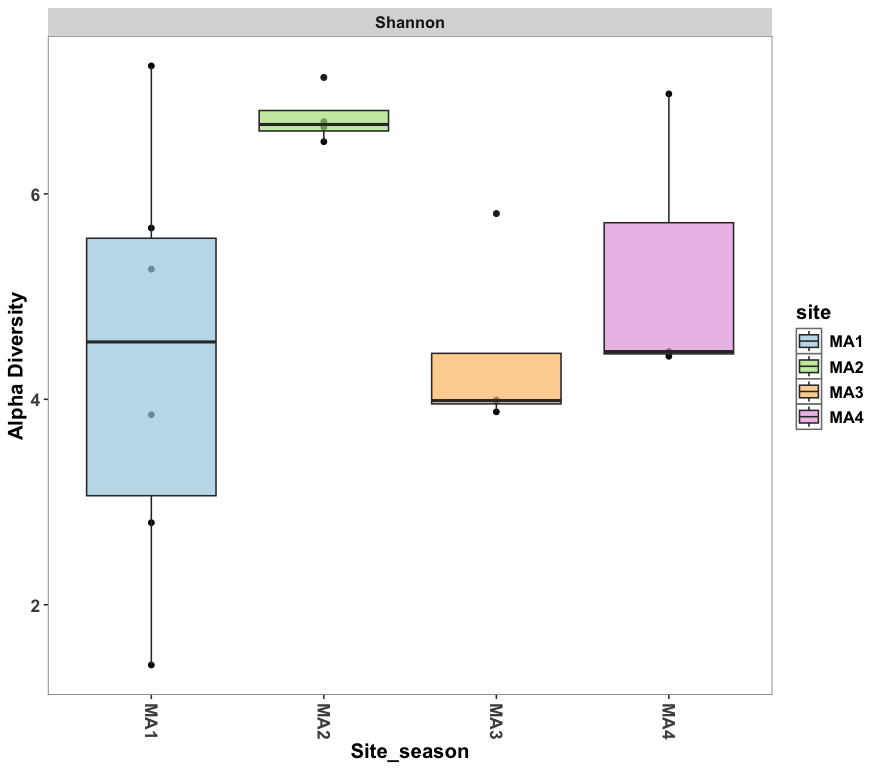


Figure S-4. Diversity Indexes based on ASVs (Shannon, Simpson, InvSimpson) applied to Microeukaryotic communities as well as average diversity for each site.


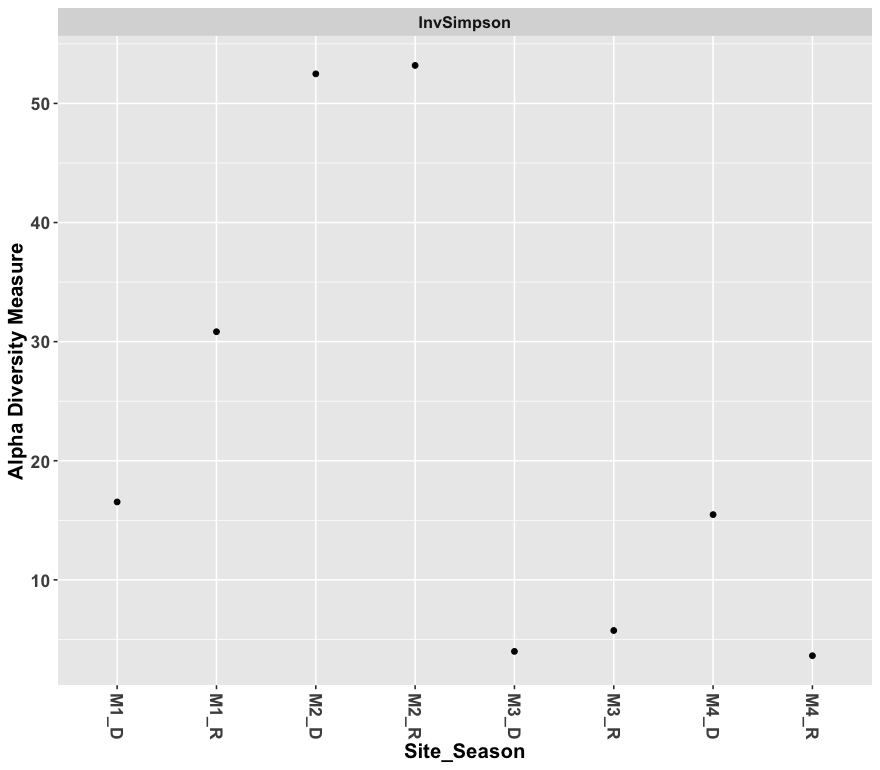

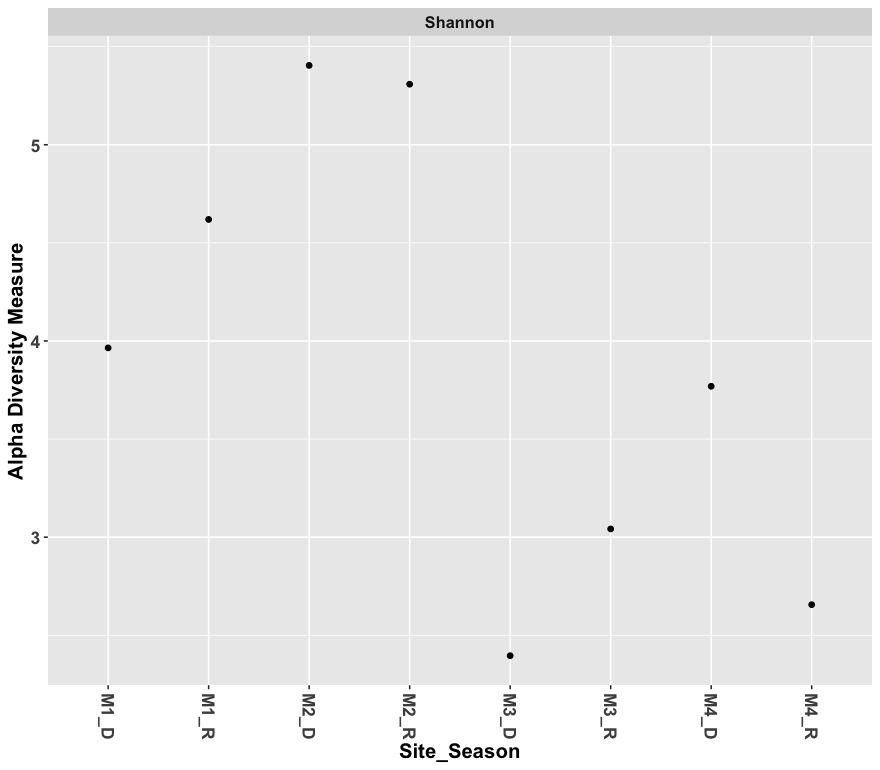

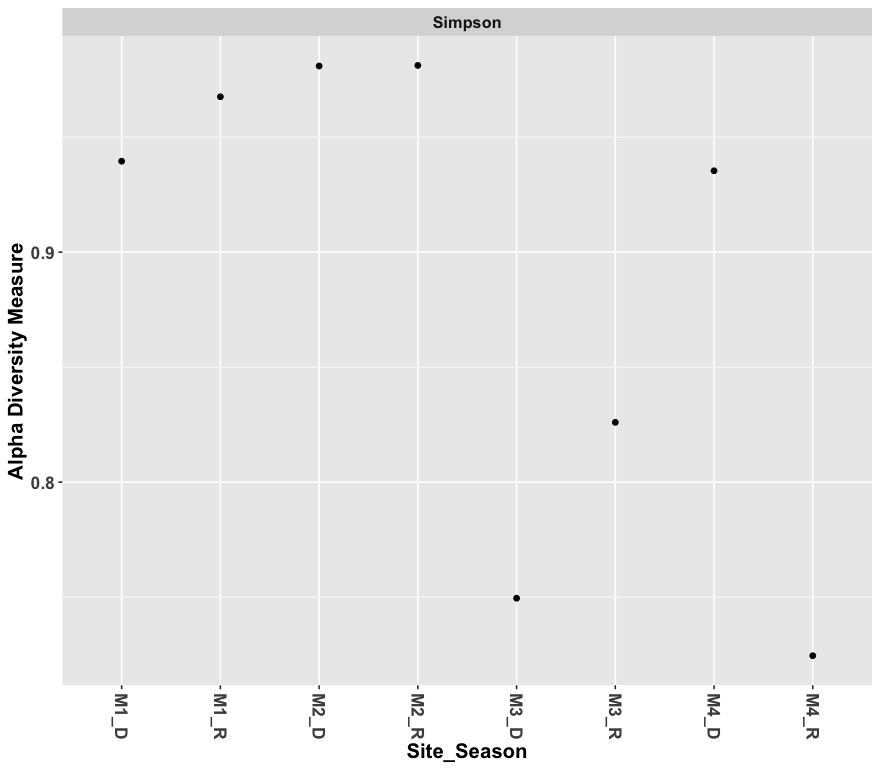


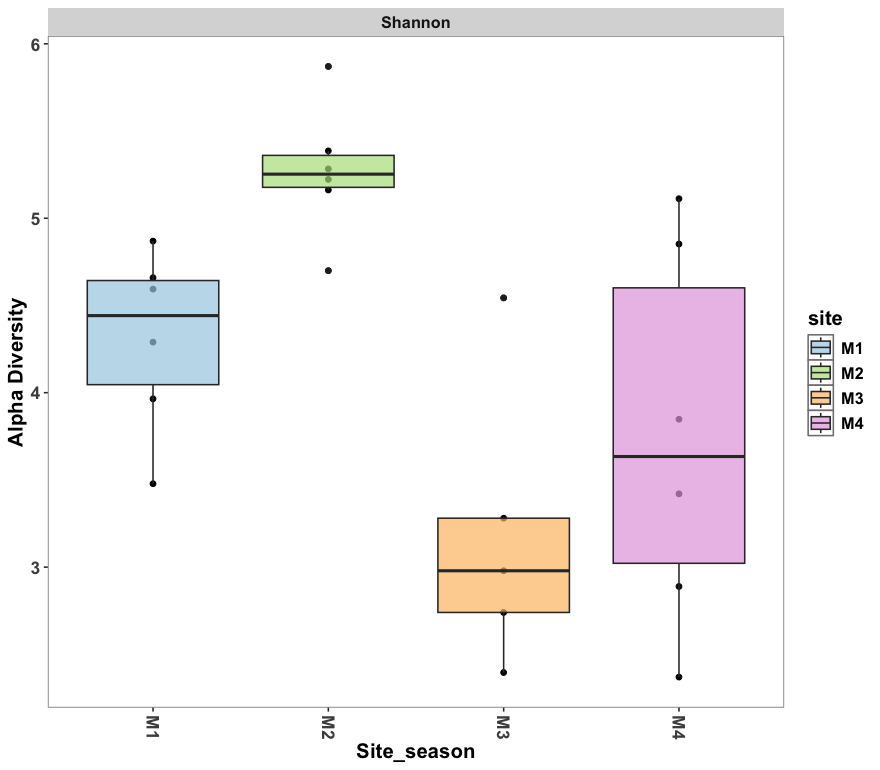


Figure S-5. Explained Variances of Principal Components for Microeukaryotic Communities


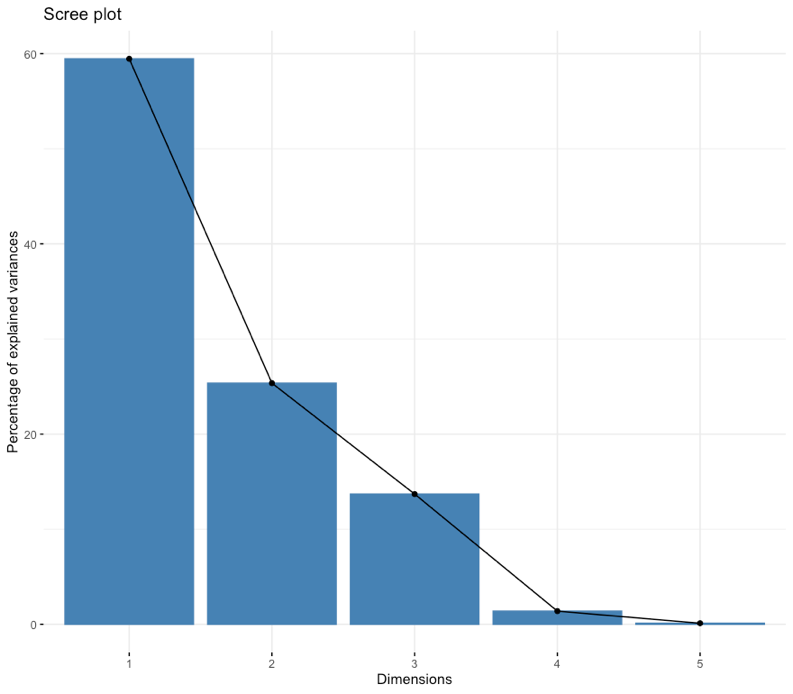


Figure S-6. Explained Variances of Principal Components for Prokaryotic Communities


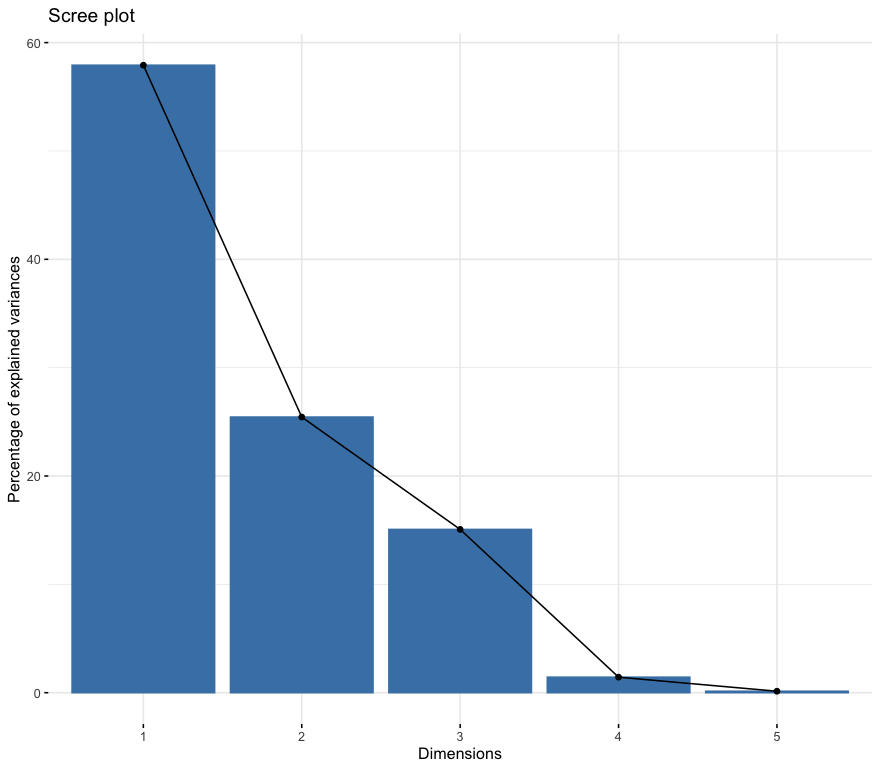


Table S-1. Prokaryotic Phyla and their relative abundance in % for all sites and seasons

| **Phylum** | **M1_D** | **M2_D** | **M3_D** | **M4_D** | **M1_R** | **M2_R** | **M3_R** | **M4_R** | **Total_Phyllum** | **Average_per_phyllum (%)** |
| --- | --- | --- | --- | --- | --- | --- | --- | --- | --- | --- |
| Proteobacteria | 40819 | 61746 | 55974 | 57339 | 10599.5 | 55382.5 | 44384.5 | 74916 | 401160.5 | 45.46 |
| Cyanobacteria | 36458 | 1059 | 187 | 108 | 88563 | 13661.5 | 521 | 747 | 141304.5 | 16.01 |
| Campilobacterota | 1143 | 1480 | 32959 | 26298 | 405.5 | 1491 | 46791.5 | 14681 | 125249 | 14.19 |
| Bacteroidota | 14626 | 22175 | 9583 | 17061 | 2231 | 12673 | 10323.5 | 12449 | 101121.5 | 11.46 |
| Verrucomicrobiota | 3307 | 5490 | 201 | 639 | 923 | 6673 | 289 | 383 | 17905 | 2.02 |
| Fusobacteriota | 238 | 320 | 6616 | 3288 | 57 | 162.5 | 2389.5 | 2412 | 15483 | 1.75 |
| Actinobacteriota | 1143 | 3838 | 239 | 244 | 1699 | 3243 | 404 | 454 | 11264 | 1.27 |
| Firmicutes | 190 | 355 | 2753 | 1955 | 150.5 | 441 | 3536 | 1815 | 11195.5 | 1.26 |
| Myxococcota | 1714 | 3020 | 99 | 69 | 1240.5 | 4034 | 224.5 | 368 | 10769 | 1.22 |
| Acidobacteriota | 2815 | 2332 | 210 | 356 | 1224.5 | 2505.5 | 211.5 | 282 | 9936.5 | 1.12 |
| Planctomycetota | 1493 | 2731 | 30 | 41 | 908.5 | 4463.5 | 112 | 127 | 9906 | 1.12 |
| Desulfobacterota | 1861 | 2111 | 938 | 1941 | 588 | 1439 | 468 | 416 | 9762 | 1.10 |
| Chloroflexi | 1784 | 983 | 50 | 40 | 778.5 | 1291 | 66 | 102 | 5094.5 | 0.57 |
| Nitrospirota | 1000 | 284 | 59 | 42 | 238.5 | 288.5 | 50 | 70 | 2032 | 0.23 |
| Gemmatimonadota | 407 | 387 | 11 | 24 | 185 | 581 | 70.5 | 136 | 1801.5 | 0.20 |
| Bdellovibrionota | 224 | 500 | 12 | 26 | 105.5 | 684 | 90 | 112 | 1753.5 | 0.19 |
| Fibrobacterota | 64 | 96 | 12 | 57 | 37.5 | 166.5 | 71.5 | 578 | 1082.5 | 0.12 |
| Spirochaetota | 61 | 195 | 42 | 162 | 58 | 156.5 | 21.5 | 12 | 708 | 0.08 |
| NB1-j | 181 | 141 | 36 | 25 | 77 | 99 | 20 | 19 | 598 | 0.06 |
| Armatimonadota | 128 | 126 | 12 | 1 | 47 | 231 | 13.5 | 6 | 564.5 | 0.06 |
| Synergistota | 0 | 19 | 121 | 389 | 0 | 1.5 | 3 | 10 | 543.5 | 0.06 |
| Latescibacterota | 170 | 86 | 8 | 4 | 88 | 75 | 10.5 | 10 | 451.5 | 0.05 |
| Unknown | 109 | 89 | 12 | 10 | 36.5 | 72.5 | 11.5 | 19 | 359.5 | 0.04 |
| Patescibacteria | 119 | 69 | 0 | 0 | 29 | 64 | 26 | 39 | 346 | 0.03 |
| Deinococcota | 20 | 179 | 0 | 6 | 12 | 77 | 4.5 | 7 | 305.5 | 0.03 |
| Hydrogenedentes | 29 | 6 | 0 | 0 | 20 | 85.5 | 28.5 | 59 | 228 | 0.02 |
| Elusimicrobiota | 94 | 37 | 7 | 17 | 21 | 27 | 8 | 5 | 216 | 0.02 |
| SAR324_clade | 17 | 21 | 13 | 57 | 3 | 33 | 18 | 27 | 189 | 0.02 |
| Nitrospinota | 108 | 11 | 0 | 0 | 33 | 11 | 0 | 0 | 163 | 0.01 |
| FCPU426 | 44 | 72 | 0 | 0 | 12 | 34.5 | 0 | 0 | 162.5 | 0.01 |
| WPS-2 | 3 | 83 | 5 | 7 | 1 | 38.5 | 3 | 3 | 143.5 | 0.01 |
| MBNT15 | 69 | 20 | 0 | 0 | 6 | 15.5 | 1.5 | 0 | 112 | 0.01 |
| Methylomirabilota | 34 | 12 | 0 | 0 | 23.5 | 20 | 0.5 | 2 | 92 | 0.01 |
| RCP2-54 | 41 | 18 | 0 | 0 | 0 | 9 | 1.5 | 0 | 69.5 | 0.007 |
| Sumerlaeota | 9 | 6 | 0 | 8 | 5.5 | 16.5 | 3 | 0 | 48 | 0.005 |
| Zixibacteria | 16 | 17 | 6 | 0 | 1.5 | 0 | 0 | 2 | 42.5 | 0.004 |
| Abditibacteriota | 6 | 6 | 0 | 0 | 3.5 | 11.5 | 0 | 0 | 27 | 0.003 |
| Deferrisomatota | 15 | 5 | 0 | 0 | 0 | 3.5 | 1 | 0 | 24.5 | 0.002 |
| Sva0485 | 9 | 6 | 0 | 0 | 0 | 3.5 | 1.5 | 0 | 20 | 0.002 |
| Entotheonellaeota | 9 | 0 | 0 | 0 | 2.5 | 3 | 1 | 0 | 15.5 | 0.001 |
| Modulibacteria | 0 | 5 | 4 | 0 | 0 | 3.5 | 1 | 0 | 13.5 | 0.001 |
| Dependentiae | 0 | 0 | 0 | 0 | 1 | 4.5 | 1 | 3 | 9.5 | 0.001 |
| Cloacimonadota | 0 | 0 | 0 | 3 | 0 | 3.5 | 2.5 | 0 | 9 | 0.001 |
| WS2 | 3 | 0 | 0 | 0 | 0 | 0 | 0 | 3 | 6 | 0.0006 |
| Margulisbacteria | 0 | 4 | 0 | 0 | 0 | 0 | 0 | 0 | 4 | 0.0004 |
| Fermentibacterota | 0 | 0 | 0 | 2 | 0 | 0 | 0 | 0 | 2 | 0.0002 |
| Calditrichota | 0 | 0 | 0 | 0 | 0 | 0 | 1.5 | 0 | 1.5 | 0.0001 |
| Dadabacteria | 0 | 0 | 0 | 0 | 0 | 0 | 1.5 | 0 | 1.5 | 0.0001 |
| LCP-89 | 0 | 0 | 0 | 0 | 1 | 0 | 0 | 0 | 1 | 0.0001 |

Table S-2. Eukaryotic Phyla and their relative abundance in % for all sites and seasons

| **Phylum** | **M1_D** | **M2_D** | **M3_D** | **M4_D** | **M1_R** | **M2_R** | **M3_R** | **M4_R** | **Total_Phyllum** | **Average_per_phyllum (%)** |
| --- | --- | --- | --- | --- | --- | --- | --- | --- | --- | --- |
| Ciliophora | 29540 | 4306.5 | 4132 | 9394.5 | 4321 | 26778.5 | 84077 | 65127.5 | 227677 | 31.29 |
| Chlorophyta | 4072 | 593.5 | 90668 | 48534 | 3402 | 1119 | 13649 | 3081 | 165118.5 | 22.69 |
| Diatomea | 29201 | 5483.5 | 267 | 9747 | 25918.5 | 10644.5 | 979 | 13606 | 95846.5 | 13.17 |
| Phragmoplastophyta | 12305 | 4690 | 1660 | 2123 | 12286 | 3785 | 2448 | 9733 | 49030 | 6.74 |
| Rotifera | 362 | 25962.5 | 2063 | 4230 | 448 | 1223 | 1215.5 | 13116 | 48620 | 6.68 |
| Arthropoda | 0 | 7566 | 56 | 701 | 17737 | 8890 | 18.5 | 335 | 35303.5 | 4.85 |
| Cercozoa | 4496 | 10705 | 74 | 111 | 1762 | 10076 | 550 | 59 | 27833 | 3.83 |
| Peronosporomycetes | 220 | 3310.5 | 334 | 854.5 | 696 | 5762 | 184.5 | 101 | 11462.5 | 1.58 |
| Nematozoa | 2303 | 2482 | 1340 | 4564 | 66 | 70.5 | 32 | 23.5 | 10881 | 1.50 |
| Annelida | 4 | 8033.5 | 32 | 66 | 678.5 | 38.5 | 13 | 20.5 | 8886 | 1.22 |
| SAR | 225 | 1635.5 | 688 | 366 | 204 | 3746.5 | 570 | 205 | 7640 | 1.05 |
| Incertae_Sedis | 151 | 2783.5 | 1903 | 517 | 116 | 581 | 769.5 | 272 | 7093 | 0.97 |
| Tardigrada | 0 | 2153.5 | 0 | 87.5 | 116.5 | 2800 | 0 | 14.5 | 5172 | 0.71 |
| Ochrophyta | 850 | 2316 | 80 | 369.5 | 235.5 | 824 | 355 | 55.5 | 5085.5 | 0.70 |
| Holozoa | 448 | 1089.5 | 99 | 174 | 860.5 | 886.5 | 99.5 | 183.5 | 3840.5 | 0.53 |
| Mollusca | 3 | 779 | 25 | 206 | 2106.5 | 362 | 0 | 43.5 | 3525 | 0.48 |
| Gastrotricha | 0 | 2575 | 0 | 0 | 0 | 0 | 0 | 0 | 2575 | 0.35 |
| Amoebozoa | 169 | 348.5 | 675 | 112 | 41.5 | 203 | 408.5 | 34 | 1991.5 | 0.27 |
| Platyhelminthes | 11 | 115 | 0 | 0 | 1465 | 17 | 0 | 0 | 1608 | 0.22 |
| Bicosoecida | 291 | 223 | 6 | 7 | 274.5 | 757.5 | 0 | 0 | 1559 | 0.21 |
| Labyrinthulomycetes | 87 | 452 | 11 | 0 | 177 | 656.5 | 7.5 | 2.5 | 1393.5 | 0.19 |
| Centrohelida | 24 | 133.5 | 0 | 0 | 10 | 809 | 4 | 1 | 981.5 | 0.13 |
| Cnidaria | 8 | 34 | 9 | 10 | 520 | 107 | 0 | 0 | 688 | 0.09 |
| Apicomplexa | 107 | 242 | 0 | 58 | 179 | 73 | 9.5 | 7.5 | 676 | 0.09 |
| LKM15 | 36 | 116.5 | 25 | 101 | 50 | 68.5 | 138 | 68.5 | 603.5 | 0.08 |
| Dinoflagellata | 34 | 234.5 | 28 | 36.5 | 104 | 41.5 | 4.5 | 0 | 483 | 0.07 |
| Rigifilida | 289 | 0 | 0 | 0 | 17.5 | 132 | 0 | 0 | 438.5 | 0.06 |
| Aphelidea | 0 | 16 | 0 | 4 | 142 | 10 | 11.5 | 201.5 | 385 | 0.05 |
| MAST-12 | 18 | 133 | 0 | 0 | 30 | 135.5 | 0 | 0 | 316.5 | 0.04 |
| Cryptophyceae | 7 | 27 | 0 | 0 | 21.5 | 65.5 | 22.5 | 0 | 143.5 | 0.02 |
| Protalveolata | 42 | 16.5 | 0 | 17 | 18 | 13 | 3.5 | 15 | 125 | 0.02 |
| MAST-3 | 35 | 27.5 | 0 | 2.5 | 0 | 59.5 | 0 | 0 | 124.5 | 0.02 |
| Kathablepharidae | 0 | 6 | 0 | 0 | 75 | 10.5 | 0 | 1.5 | 93 | 0.01 |
| Breviatea | 0 | 44 | 0 | 0 | 0 | 1.5 | 0 | 1 | 46.5 | 0.01 |
| Schizoplasmodiida | 17 | 16.5 | 0 | 0 | 5.5 | 0 | 1.5 | 0 | 40.5 | 0.01 |
| Porifera | 12 | 8.5 | 0 | 2 | 8 | 7 | 1.5 | 0 | 39 | 0.01 |
| Apusomonadidae | 6 | 6 | 0 | 0 | 0 | 21 | 3 | 0 | 36 | 0.00 |
| Preaxostyla | 0 | 0 | 0 | 0 | 0 | 0 | 33.5 | 0 | 33.5 | 0.00 |
| Lophophorata | 0 | 22.5 | 0 | 9.5 | 0 | 0 | 0 | 0 | 32 | 0.00 |
| Heterolobosea | 15 | 0 | 0 | 6.5 | 0 | 0 | 0 | 0 | 21.5 | 0.00 |
| Protosporangiida | 0 | 20.5 | 0 | 0 | 0 | 0 | 0 | 0 | 20.5 | 0.00 |
| Retaria | 0 | 2 | 15 | 3 | 0 | 0 | 0 | 0 | 20 | 0.00 |
| Ctenophora | 0 | 7 | 0 | 0 | 0 | 9.5 | 0 | 0 | 16.5 | 0.00 |
| Bangiales | 0 | 0 | 0 | 0 | 10 | 1 | 0 | 0 | 11 | 0.00 |
| Ancyromonadida | 0 | 2.5 | 0 | 0 | 0 | 7.5 | 0 | 0 | 10 | 0.00 |
| Gracilipodida | 0 | 2.5 | 4 | 0 | 0 | 3 | 0 | 0 | 9.5 | 0.00 |
| Scalidophora | 0 | 9.5 | 0 | 0 | 0 | 0 | 0 | 0 | 9.5 | 0.00 |
| Archamoebae | 0 | 6 | 0 | 0 | 0 | 0 | 0 | 0 | 6 | 0.00 |
| Picozoa | 0 | 5 | 0 | 0 | 0 | 0 | 0 | 0 | 5 | 0.00 |
| Nucleariidae_and_Fonticula_group | 0 | 0 | 0 | 0 | 4 | 0 | 0 | 0 | 4 | 0.00 |
| Protosteliida | 0 | 0 | 0 | 0 | 4 | 0 | 0 | 0 | 4 | 0.00 |
| Tunicata | 0 | 0 | 0 | 4 | 0 | 0 | 0 | 0 | 4 | 0.00 |

Table S-3. Top 50 Prokaryotic Genus and their relative abundance in % for all sites and seasons

| **Genus** | **M1_D** | **M2_D** | **M3_D** | **M4_D** | **M1_R** | **M2_R** | **M3_R** | **M4_R** |  | **Average %** |
| --- | --- | --- | --- | --- | --- | --- | --- | --- | --- | --- |
| Chloroplast | 36350 | 952 | 185 | 100 | 88355.5 | 13315.5 | 443 | 679 | 140380 | 18.07 |
| Arcobacter | 965 | 1261 | 30017 | 22373 | 350.5 | 1304 | 41650 | 13535 | 111455.5 | 14.35 |
| Acinetobacter | 1242 | 1471 | 13443 | 17107 | 540 | 2228.5 | 17481.5 | 30989 | 84502 | 10.88 |
| Aeromonas | 827 | 1216 | 21072 | 8387 | 183.5 | 883 | 6080.5 | 10777 | 49426 | 6.36 |
| Flavobacterium | 6729 | 9030 | 635 | 410 | 299 | 2547 | 661.5 | 1789 | 22100.5 | 2.84 |
| Pseudomonas | 11422 | 2240 | 2871 | 2031 | 147.5 | 520 | 1078.5 | 1364 | 21674 | 2.79 |
| Rhodoferax | 3650 | 6030 | 415 | 706 | 350.5 | 4912 | 259.5 | 276 | 16599 | 2.14 |
| Bacteroides | 154 | 271 | 3480 | 5512 | 26.5 | 113.5 | 2803 | 2525 | 14885 | 1.92 |
| Acidovorax | 945 | 1569 | 1660 | 3617 | 268.5 | 1642 | 1125.5 | 2841 | 13668 | 1.76 |
| Pseudarcobacter | 108 | 164 | 1813 | 3487 | 48 | 168.5 | 4646 | 1062 | 11496.5 | 1.48 |
| Leptotrichia | 164 | 204 | 4740 | 2968 | 16.5 | 47.5 | 456 | 422 | 9018 | 1.16 |
| Comamonas | 286 | 442 | 2054 | 2487 | 51.5 | 343 | 1534 | 1420 | 8617.5 | 1.11 |
| Tolumonas | 116 | 201 | 1171 | 2094 | 64 | 201 | 1428.5 | 3056 | 8331.5 | 1.07 |
| Rhizobacter | 802 | 3494 | 84 | 101 | 632.5 | 2847.5 | 107.5 | 135 | 8203.5 | 1.06 |
| Macellibacteroides | 119 | 169 | 2226 | 2753 | 15.5 | 74.5 | 1341.5 | 911 | 7609.5 | 0.98 |
| Enhydrobacter | 139 | 186 | 523 | 2127 | 55 | 252 | 788 | 3522 | 7592 | 0.98 |
| Zoogloea | 148 | 226 | 384 | 2259 | 65 | 256 | 983.5 | 2947 | 7268.5 | 0.94 |
| Cloacibacterium | 40 | 66 | 311 | 806 | 50.5 | 185.5 | 1624 | 3883 | 6966 | 0.90 |
| SC-I-84 | 1113 | 2298 | 53 | 53 | 557 | 2035.5 | 92.5 | 106 | 6308 | 0.81 |
| Dechloromonas | 181 | 1441 | 192 | 750 | 35 | 1556.5 | 401 | 605 | 5161.5 | 0.66 |
| Lacunisphaera | 914 | 1026 | 126 | 519 | 221 | 1767 | 113 | 136 | 4822 | 0.62 |
| Hypnocyclicus | 26 | 40 | 862 | 128 | 35 | 86 | 1747.5 | 1863 | 4787.5 | 0.62 |
| Sphaerotilus | 56 | 846 | 18 | 29 | 29 | 1592.5 | 132.5 | 1298 | 4001 | 0.51 |
| Propionivibrio | 153 | 384 | 615 | 1657 | 9.5 | 274 | 297 | 568 | 3957.5 | 0.51 |
| Undibacterium | 915 | 2414 | 80 | 84 | 29 | 320.5 | 35 | 42 | 3919.5 | 0.50 |
| Cellvibrio | 239 | 1863 | 72 | 67 | 131.5 | 1186.5 | 101.5 | 118 | 3778.5 | 0.49 |
| Ellin6067 | 1148 | 669 | 28 | 16 | 452 | 755 | 52 | 58 | 3178 | 0.41 |
| Prevotella | 12 | 17 | 321 | 467 | 5.5 | 37 | 1759 | 422 | 3040.5 | 0.39 |
| Hydrogenophaga | 53 | 450 | 113 | 131 | 36.5 | 718 | 1076 | 444 | 3021.5 | 0.39 |
| Candidatus_Accumulibacter | 199 | 805 | 262 | 836 | 67 | 555.5 | 103 | 184 | 3011.5 | 0.39 |
| env.OPS_17 | 380 | 1715 | 37 | 23 | 77 | 710 | 26 | 40 | 3008 | 0.39 |
| Novosphingobium | 471 | 1228 | 49 | 52 | 84 | 895 | 82 | 120 | 2981 | 0.38 |
| Paludibacter | 32 | 123 | 230 | 1219 | 11 | 78 | 307 | 936 | 2936 | 0.38 |
| OM60(NOR5)_clade | 496 | 1254 | 53 | 42 | 124.5 | 734.5 | 37.5 | 60 | 2801.5 | 0.36 |
| Rhodobacter | 185 | 803 | 124 | 590 | 90.5 | 866 | 42 | 67 | 2767.5 | 0.36 |
| Aquabacterium | 96 | 699 | 109 | 195 | 21.5 | 766.5 | 528.5 | 300 | 2715.5 | 0.35 |
| Thauera | 78 | 156 | 490 | 838 | 10.5 | 98.5 | 388.5 | 645 | 2704.5 | 0.35 |
| Ferruginibacter | 740 | 849 | 23 | 15 | 168 | 739.5 | 5 | 12 | 2551.5 | 0.33 |
| Rivicola | 29 | 26 | 83 | 293 | 28 | 124.5 | 169 | 1688 | 2440.5 | 0.31 |
| Arenimonas | 208 | 677 | 33 | 19 | 169.5 | 776 | 223 | 325 | 2430.5 | 0.31 |
| Vitreoscilla | 61 | 111 | 780 | 540 | 12 | 61.5 | 478 | 386 | 2429.5 | 0.31 |
| Sulfuritalea | 544 | 1029 | 19 | 23 | 109.5 | 599 | 33.5 | 50 | 2407 | 0.31 |
| BIrii41 | 375 | 572 | 41 | 23 | 313 | 954 | 26 | 49 | 2353 | 0.30 |
| Vicinamibacteraceae | 509 | 523 | 46 | 23 | 235 | 586.5 | 50 | 64 | 2036.5 | 0.26 |
| Opitutus | 408 | 558 | 0 | 27 | 82.5 | 792 | 0 | 30 | 1897.5 | 0.24 |
| Geobacter | 489 | 564 | 89 | 92 | 167 | 385.5 | 61.5 | 34 | 1882 | 0.24 |
| Williamwhitmania | 33 | 84 | 369 | 1257 | 1.5 | 7 | 29.5 | 74 | 1855 | 0.24 |
| Nitrospira | 864 | 262 | 52 | 35 | 209 | 267.5 | 50 | 70 | 1809.5 | 0.23 |
| Desulfobulbus | 93 | 302 | 243 | 794 | 23 | 204 | 62.5 | 62 | 1783.5 | 0.23 |
| Enterobacter | 53 | 101 | 623 | 307 | 10.5 | 82 | 273.5 | 324 | 1774 | 0.23 |

Table S-4. Top 50 Eukaryotic Genus and their relative abundance in % for all sites and seasons

| **Genus** | **M1_D** | **M2_D** | **M3_D** | **M4_D** | **M1_R** | **M2_R** | **M3_R** | **M4_R** |  | **Average %** |
| --- | --- | --- | --- | --- | --- | --- | --- | --- | --- | --- |
| Chlorophyceae | 4.41094437 | 0.23763811 | 87.7884333 | 39.2152423 | 3.75143581 | 0.70231539 | 35.0025257 | 1.8618999 | 172.970435 | 21.62 |
| Telotrochidium | 0 | 0.00507155 | 0.01174283 | 0.03862521 | 1.06066654 | 0.20252265 | 26.0627974 | 58.9902275 | 86.3716538 | 10.80 |
| Adinetida | 0.14013796 | 36.1057779 | 2.00998141 | 5.58933411 | 0.48145463 | 0.72126488 | 3.23159546 | 12.5257265 | 60.8052728 | 7.60 |
| Magnoliophyta | 8.67086627 | 6.11918131 | 1.54809668 | 2.60054208 | 18.9893526 | 7.76573696 | 5.38510621 | 9.11568151 | 60.1945636 | 7.52 |
| Nitzschia | 2.77962965 | 0.54772686 | 0.10959977 | 12.4952551 | 1.09651088 | 0.46544679 | 2.51508787 | 12.9627764 | 32.9720333 | 4.12 |
| Trebouxiophyceae | 1.00681642 | 0.36804927 | 0.93649085 | 25.3787601 | 0.78531686 | 1.09077989 | 1.2508973 | 1.07991153 | 31.8970222 | 3.99 |
| Bacillariophyceae | 24.8602022 | 0.06520558 | 0.09687836 | 0.26504885 | 2.12377702 | 0.16936105 | 0.00531731 | 0 | 27.5857903 | 3.45 |
| Harpacticoida | 0 | 4.27821047 | 0 | 0 | 0.16129952 | 20.9995855 | 0 | 0.00239874 | 25.4414942 | 3.18 |
| Paramecium | 3.83950802 | 0.13403369 | 0.72511987 | 1.47974507 | 0.37880948 | 0.05803281 | 13.422221 | 1.96360636 | 22.0010763 | 2.75 |
| Pseudourostyla | 11.5919944 | 0.77304836 | 0.05284274 | 0.13119252 | 1.23092715 | 8.13643631 | 0 | 0 | 21.9164415 | 2.74 |
| Podocopida | 0 | 0 | 0 | 0 | 19.1848672 | 0.02131817 | 0.00265866 | 0 | 19.208844 | 2.40 |
| Navicula | 5.52932693 | 0.85709111 | 0.01859282 | 0.06193352 | 10.392414 | 1.80138568 | 0.01595193 | 0.01055444 | 18.6872505 | 2.34 |
| Haptoria | 5.48170723 | 0.44339794 | 0.85233389 | 1.45776866 | 1.32705514 | 7.13211346 | 0.02126924 | 0.00095949 | 16.7166051 | 2.09 |
| Acari | 0 | 6.16772324 | 0.02446423 | 0.89304147 | 9.18999943 | 0.03197726 | 0.00265866 | 0.00191899 | 16.3117833 | 2.04 |
| Tetrahymena | 10.6695329 | 0.25140373 | 0.0733927 | 0.24040863 | 0.2940865 | 3.74489252 | 0.00664664 | 0.01247343 | 15.2928371 | 1.91 |
| Sorodiplophrys | 0.30612661 | 2.36986053 | 0.6732557 | 0.48747677 | 0.33237477 | 8.87428199 | 1.51543349 | 0.19669646 | 14.7555063 | 1.84 |
| Haplotaxida | 0.00544225 | 11.4805289 | 0.03131422 | 0.08790565 | 1.10547196 | 0.07816664 | 0.02126924 | 0.01966965 | 12.8297685 | 1.60 |
| Parachela | 0 | 3.12044919 | 0 | 0.11654158 | 0.18981206 | 6.63232072 | 0 | 0.01391268 | 10.0730362 | 1.26 |
| Melosira | 0.57279691 | 0 | 0 | 0 | 8.96841625 | 0.50216143 | 0 | 0 | 10.0433746 | 1.26 |
| Zygnematophyceae | 8.03004123 | 0.03115378 | 0.05088561 | 0.07525256 | 0.65741774 | 0.34109078 | 0 | 0.00383798 | 9.18967967 | 1.15 |
| Diplogasterida | 0.05170138 | 0.01593914 | 1.28877581 | 5.99556476 | 0 | 0 | 0.08507697 | 0.02254813 | 7.45960619 | 0.93 |
| Rhogostoma | 0.00544225 | 0.82955986 | 0 | 0.00599357 | 0.16129952 | 4.12506662 | 0.10634621 | 0 | 5.23370803 | 0.65 |
| Blastocystis | 0.10476333 | 0 | 1.86221744 | 0.68526448 | 0.06680081 | 0 | 2.03254194 | 0.21924459 | 4.97083259 | 0.62 |
| Spirostomum | 0 | 0.47237819 | 0 | 0 | 0.05946902 | 0.01065909 | 4.03052136 | 0.00815571 | 4.58118336 | 0.57 |
| Caenogastropoda | 0 | 0 | 0 | 0 | 3.39869494 | 0.84798958 | 0 | 0 | 4.24668452 | 0.53 |
| Pirsonia | 0 | 3.70874842 | 0 | 0 | 0 | 0.41215136 | 0.00930529 | 0.04173803 | 4.1719431 | 0.52 |
| Ploimida | 0.35238575 | 1.238906 | 0.00880712 | 0.04328687 | 0.24846643 | 2.08089063 | 0 | 0.03070384 | 4.00344663 | 0.50 |
| Entodinium | 3.30616743 | 0.02970476 | 0.31216362 | 0.23374911 | 0.00896108 | 0 | 0 | 0.00143924 | 3.89218525 | 0.49 |
| Planothidium | 1.00137417 | 0.28328201 | 0 | 0 | 2.02927831 | 0.56730029 | 0 | 0 | 3.88123478 | 0.49 |
| Pinnularia | 0.10884502 | 0.07100163 | 0 | 0 | 3.36855311 | 0.27476757 | 0.01329328 | 0 | 3.83646061 | 0.48 |
| Chaetonotida | 0 | 3.73120811 | 0 | 0 | 0 | 0 | 0 | 0 | 3.73120811 | 0.47 |
| Monhysterida | 0 | 3.40155769 | 0 | 0 | 0 | 0 | 0 | 0 | 3.40155769 | 0.43 |
| Pythium | 0.02040844 | 0.93896033 | 0.268128 | 0.08191208 | 0.096128 | 1.82033517 | 0.07843033 | 0.02254813 | 3.32685048 | 0.42 |
| Holosticha | 0 | 0.06955262 | 0 | 0 | 0.14826522 | 3.04376147 | 0 | 0 | 3.26157931 | 0.41 |
| Mononchida | 3.08167458 | 0 | 0.00978569 | 0.03662735 | 0 | 0 | 0 | 0 | 3.12808763 | 0.39 |
| Hypotrichia | 0.07483095 | 0.02101069 | 0.03033565 | 0.19778771 | 0 | 2.54278439 | 0.00930529 | 0 | 2.87605468 | 0.36 |
| Pinophyta | 0.01360563 | 0.563666 | 0.0254428 | 0.15183703 | 0.11567945 | 0.29253272 | 1.11929386 | 0.18086479 | 2.46292229 | 0.31 |
| Stenostomidae | 0.01496619 | 0.10940047 | 0 | 0 | 2.19139247 | 0.0331616 | 0 | 0 | 2.34892074 | 0.29 |
| Heteromita | 0.07483095 | 0.73827205 | 0.00587142 | 0.04062306 | 0.09694264 | 1.26961568 | 0.06513705 | 0.00671646 | 2.29800932 | 0.29 |
| Vorticella | 0.33197731 | 0.81506973 | 0.21332811 | 0.39224566 | 0.04724935 | 0.39793924 | 0 | 0 | 2.19780941 | 0.27 |
| Scuticociliatia | 0 | 0.34124253 | 0 | 0 | 0.00977573 | 0.01776514 | 1.50214022 | 0.03598106 | 1.90690468 | 0.24 |
| Ulvophyceae | 0.08571545 | 0.13330918 | 0 | 0.02796998 | 0.74784323 | 0.79824717 | 0.02791588 | 0.01103419 | 1.83203509 | 0.23 |
| Paraphysomonas | 0.12517177 | 0.44702047 | 0.01174283 | 0.02796998 | 0.0887962 | 1.09077989 | 0 | 0.00095949 | 1.79244063 | 0.22 |
| BOLA868 | 0.18775766 | 0.32023184 | 0.00587142 | 0 | 0.03258576 | 0.25581808 | 0.36024779 | 0.00143924 | 1.16395179 | 0.15 |
| LKM15 | 0.04898026 | 0.16881 | 0.02446423 | 0.13452228 | 0.0814644 | 0.16225499 | 0.36689442 | 0.0657254 | 1.05311599 | 0.13 |
| Cyphoderia | 0 | 0.79478355 | 0 | 0 | 0.18736813 | 0.03434595 | 0 | 0 | 1.01649763 | 0.13 |
| Labyrinthuloides | 0.08843658 | 0.26516935 | 0 | 0 | 0.11567945 | 0.50808314 | 0.00398798 | 0 | 0.98135651 | 0.12 |
| Gomphonema | 0.25850692 | 0.0449194 | 0.00587142 | 0.00932333 | 0.52463076 | 0.08764138 | 0 | 0.04221778 | 0.97311098 | 0.12 |
| Protaspis | 0.19319991 | 0.22821953 | 0 | 0 | 0 | 0.54716646 | 0 | 0 | 0.96858589 | 0.12 |

Table S-5. Variance of the Principal Component Analysis and Cumulative Proportion for ASVs in Microbial Communities.

| Principal Components for microeukaryotic communities | | | | | |
| --- | --- | --- | --- | --- | --- |
|  | PC1 | PC2 | PC3 | PC4 | PC5 |
| Proportion of Variance | 0.4924 | 0.2728 | 0.0975 | 0.0869 | 0.0279 |
| Cumulative Proportion | 0.4924 | 0.7653 | 0.8628 | 0.9498 | 0.9777 |
|  | | | | | |
| Principal Components for prokaryotic communities | | | | | |
|  | PC1 | PC2 | PC3 | PC4 | PC5 |
| Proportion of Variance | 0.4674 | 0.1256 | 0.0672 | 0.0584 | 0.0525 |
| Cumulative Proportion | 0.4674 | 0.5930 | 0.6603 | 0.7187 | 0.7712 |
